# Computationally efficient joint species distribution modeling of big spatial data

**DOI:** 10.1002/ecy.2929

**Published:** 2019-12-20

**Authors:** Gleb Tikhonov, Li Duan, Nerea Abrego, Graeme Newell, Matt White, David Dunson, Otso Ovaskainen

**Affiliations:** ^1^ Organismal and Evolutionary Biology Research Programme University of Helsinki P.O. Box 65 FI‐00014 Helsinki Finland; ^2^ Computational Systems Biology Group Department of Computer Science Aalto University P.O. Box 11000 FI‐00076 Espoo Finland; ^3^ Department of Statistics University of Florida P.O. Box 118545 Gainesville Florida 32611 USA; ^4^ Faculty of Biological and Environmental Sciences University of Helsinki P.O. Box 65 FI‐00014 Helsinki Finland; ^5^ Biodiversity Division Department of Environment, Land, Water & Planning Arthur Rylah Institute for Environmental Research 123 Brown Street Heidelberg Victoria 3084 Australia; ^6^ Department of Statistical Science Duke University P.O. Box 90251 Durham North Carolina USA; ^7^ Centre for Biodiversity Dynamics Department of Biology Norwegian University of Science and Technology N‐7491 Trondheim Norway

**Keywords:** community modeling, ecological communities, Gaussian process, hierarchical modeling of species communities, joint species distribution model, latent factors, spatial statistics

## Abstract

The ongoing global change and the increased interest in macroecological processes call for the analysis of spatially extensive data on species communities to understand and forecast distributional changes of biodiversity. Recently developed joint species distribution models can deal with numerous species efficiently, while explicitly accounting for spatial structure in the data. However, their applicability is generally limited to relatively small spatial data sets because of their severe computational scaling as the number of spatial locations increases. In this work, we propose a practical alleviation of this scalability constraint for joint species modeling by exploiting two spatial‐statistics techniques that facilitate the analysis of large spatial data sets: Gaussian predictive process and nearest‐neighbor Gaussian process. We devised an efficient Gibbs posterior sampling algorithm for Bayesian model fitting that allows us to analyze community data sets consisting of hundreds of species sampled from up to hundreds of thousands of spatial units. The performance of these methods is demonstrated using an extensive plant data set of 30,955 spatial units as a case study. We provide an implementation of the presented methods as an extension to the hierarchical modeling of species communities framework.

## Introduction

Increased interest in large‐scale ecological processes, such as those triggered by the ongoing global change, requires the use of spatially extensive data. High‐resolution data sets covering large spatial scales are increasingly available to the scientific community, making more extensive analyses possible (Graham et al. 2004, Guralnick et al. 2007, Franklin et al. 2017). One of the key challenges is that most traditional statistical frameworks used by ecologists are computationally intractable for large data sets when the researcher aims to account for the spatial nature of the data. This leads to inefficiencies, with the data either being subsampled, or the statistical method being compromised, for example, by ignoring the spatial dependency. This illustrates the urgent need for robust statistical frameworks that enable efficient use of big spatial data for accurately describing and predicting patterns of global biodiversity.

A recent focus in statistical ecology has led to the development of approaches that jointly model the dynamics and distributions of entire species communities or ecosystems (see D’Amen et al. 2017 and references therein). In particular, joint species distribution models (JSDMs) have emerged as efficient tools for modeling data on large numbers of species, typically incorporating species dependencies through latent factors (Clark et al. 2014, Warton et al. 2015, Ovaskainen et al. 2017). Spatial extensions of JSDMs (Thorson et al. 2015, Ovaskainen et al. 2016) borrow from multivariate spatial statistics by allowing latent factors to be spatially autocorrelated (Latimer et al. 2009). These works exploit the linear model of coregionalization approach to account not only for spatial autocorrelation within each species, but also for spatial cross‐correlation among species (Genton and Kleiber 2015). However, even for the case of single‐species distribution modeling, classical spatial statistics methods require the inversion of a dense spatial covariance matrix and hence are not feasible for a large data set involving thousands of spatial locations (Banerjee et al. 2008). Because the computational burden of multivariate spatial modeling is even higher, enabling the use of JSDMs for big spatial data remains a key challenge in statistical ecology (Ovaskainen et al. 2016).

The aim of this study is to alleviate this computational impasse such that spatial JSDMs can be applied to global high‐resolution species data sets and earth observation data. To do so, we consider two spatial statistics techniques: Gaussian predictive process (GPP; Banerjee et al. 2008) and nearest‐neighbor Gaussian process (NNGP; Datta et al. 2016). Both methods approximate the full Gaussian process (GP) in a manner that enables modeling spatially extensive data (Banerjee et al. 2008), but they are based upon fundamentally different underlying mathematical constructions, leading to important differences in their properties. We implement both the GPP and NNGP approaches in the latent factor structure of hierarchical model of species communities (HMSC), which is a Bayesian JSDM framework that enables the joint analysis of data on species occurrences, environmental covariates, species traits, and phylogenetic relationships (Ovaskainen et al. 2017). We present a block Gibbs sampler that enables computationally efficient sampling from the posterior distribution of model parameters. To demonstrate the utility of the HMSC models augmented with a GPP or a NNGP, we compare their predictive and computational performances to a GP‐based spatial HMSC model as well as to a nonspatial model.

## Materials and Methods

### Hierarchical modeling of species communities (HMSC)

Our work extends HMSC proposed by Ovaskainen et al. (2016) to large, spatially explicit, ecological data sets. We consider a set of species surveyed across a set of spatial locations, hereafter called sites. We denote the sites by index $i=1,\ldots n_{y}$, and the species by index $j=1,\ldots n_{s}$, where $n_{y}$ is the number of sites and $n_{s}$ is the number of species. We denote the spatial coordinates of site i by $s_{i}=\left[s_{i1},\ldots ,s_{in_{d}}\right]$, with typically $n_{d}=2$ for ecological data. To accommodate various types of data (e.g., presence–absence, count, biomass, or timing), we follow the generalized linear modeling paradigm and model the observations as $y_{\mathrm{ij}}∼D_{j}\left(L_{\mathrm{ij}},\sigma_{j}^{2}\right)$, where $D_{j}$ is a statistical distribution compatible with the particular type of measured data, so that commonly the expectation $E\left(y_{\mathrm{ij}}\right)=g_{j}^{-1}\left(L_{\mathrm{ij}}\right)$ is parametrized by the latent variable $L_{\mathrm{ij}}$ transformed with $g_{j}$ link function, and $\sigma_{j}^{2}$ is the additional variance parameter of distribution $D_{j}$, which is omitted for certain distributions, for example, Bernoulli. The latent variable is modeled as a combination of a linear regression and spatially structured residuals:

(1) $$L_{\mathrm{ij}}=\sum_{k=1}^{n_{c}}x_{\mathrm{ik}}\beta_{\mathrm{kj}}+\varepsilon_{\mathrm{ij}},\;\text{where}\;\;\;\;\;\varepsilon_{\mathrm{ij}}=\sum_{h=1}^{n_{f}}\eta_{\mathrm{ih}}\lambda_{\mathrm{hj}}.$$

In the linear regression part, the index κ runs over a set of $n_{c}$ covariates, $x_{\mathrm{ik}}$ is the covariate k for site i, and $\beta_{\mathrm{kj}}$ is the response of species j to this covariate. The intercept is included by setting $x_{i1}=1$ for all sites i, so that the number of included environmental covariates is $n_{c}-1$. To exploit potentially available information on species‐specific traits and phylogenetic relationships, we follow the approach of Ovaskainen et al. (2017) (see Appendix S1).

In this work, our particular focus is on the term $\varepsilon_{\mathrm{ij}}$ of Eq. 1. It models species associations through a linear combination of $n_{f}$ site‐specific latent factors $\eta_{\mathrm{ih}}$ with species‐specific latent loadings $\lambda_{\mathrm{jh}}$. With the classic factor analysis assumption of factors $\eta_{\mathrm{ih}}$ having standard Gaussian prior, the species‐to‐species covariance structure (at the scale of the model’s latent variable $L_{\mathrm{ij}}$) is given by $\varepsilon_{i\cdot }∼N\left(0,\Omega \right)$, where the species‐to‐species covariance matrix can be written as Ω=ΛTΛ, and Λ is the matrix of latent loadings $\lambda_{\mathrm{hj}}$ (Ovaskainen et al. 2017). For many practical applications with large communities, the effective number of independent factors is much smaller than the total number of observed species $n_{f}\ll n_{s}$, which leads to a low‐rank approximation of Ω. Following the notation from Ovaskainen et al. (2017), the species‐to‐species association matrix is defined as the correlation‐scaled covariance matrix Ω **.** We assume the sparse Bayesian infinite factor model (Bhattacharya and Dunson 2011) for the latent loadings, so theoretically the number of factors is infinite, but in practice their number is truncated either by omitting negligible ones or by setting it to a value chosen by the user.

The spatial structure is added to the latent factors $\eta_{\cdot h}$ by assuming a Gaussian process (GP) prior $w_{h}\left(s\right)∼\text{GP}\left(0,k_{h}\left(s_{1},s_{2}\right)\right)$ (Banerjee et al. 2014). This implies that the hth latent factor a priori follows the multivariate Gaussian distribution $\eta_{\cdot h}∼N\left(0_{n_{y}\times 1},K_{\mathrm{SS}}^{\left(h\right)}\right)$, where the $K_{\mathrm{SS}}^{\left(h\right)}$ is the covariance matrix for sites $S=\left\{s_{1},\ldots ,s_{n_{y}}\right\}$, with covariance ${\left[K_{\mathrm{SS}}^{\left(h\right)}\right]}_{i_{1}i_{2}}=k_{h}\left(s_{i_{1}},s_{i_{2}}\right)$ for the pair of sites $i_{1}$ and $i_{2}$. At the level of the matrix of residuals E, this implies the spatial cross‐covariance structure $\text{vec}\left(E\right)∼N\left(0_{n_{y}n_{s}\times 1},\sum_{h=1}^{n_{f}}\left(\Lambda_{h\cdot }^{T}\Lambda_{h\cdot }⊗K_{\mathrm{SS}}^{\left(h\right)}\right)\right)$. It also implies marginal single‐species covariance structures $\varepsilon_{\cdot j}∼N\left(0_{n_{y}\times 1},\sum_{h=1}^{n_{f}}\left(\lambda_{\mathrm{hj}}^{2}K_{\mathrm{SS}}^{\left(h\right)}\right)\right)$. Here we assume the exponential covariance function $k_{h}\left(s_{1},s_{2}\right)=\exp\left(-\alpha_{h}^{-1}\left|\right|s_{1}-s_{2}\left|\right|\right)$, parametrized by a single spatial range parameter $\alpha_{h}$, which is learned during model fitting. This covariance function implies stationarity and isotropy, and it has been applied in previous work on spatial JSDMs (Thorson et al. 2015, Ovaskainen et al. 2016).

### Approximate models for big spatial data

The motivation of this work is the computational complexity of the GP‐based HMSC—the Gibbs Markov chain Monte Carlo (MCMC) updates are of the order $O\left(n_{y}^{3}\right)$ in processing time and $O\left(n_{y}^{2}\right)$ in memory storage. This means that the model is practically infeasible to apply to data sets even with moderately large numbers of sites, such as $n_{y}$ being in the order of thousands. In this study we explore two approaches from spatial statistics that has been shown to enable efficient modeling of big spatial data sets: Gaussian predictive process (GPP; Banerjee et al. 2008, Finley et al. 2015) and nearest‐neighbor Gaussian process (NNGP; Datta et al. 2016), although we note that various alternative techniques are also available (Heaton et al. 2018). We summarize the GPP and NNGP approaches briefly and provide more detailed descriptions in Appendix S1.

The GPP $\tilde{w}\left(s\right)$ assumes that all information on the original GP $w\left(s\right)$ can be summarized by a multivariate Gaussian distribution at m “knot” locations $S^{*}=\left\{s_{1}^{*},\ldots ,s_{m}^{*}\right\}$ (a.k.a. inducing points). Therefore, the value of the GPP at any location $s_{0}$ can be reconstructed as $\tilde{w}\left(s_{0}\right)=E\left(w\left(s_{0}\right)|w^{*}\right)=K_{s_{0}S^{*}}K_{S^{*}S^{*}}^{-1}w^{*}$, where $w^{*}={\left[w\left(s_{1}^{*}\right),\ldots ,w\left(s_{m}^{*}\right)\right]}^{T}$ denotes the vector of the original GP values at the knot locations $S^{*}$. With this definition, it follows that $\tilde{w}$ is itself a GP, where the covariance function is nonstationary but leads to a factorizable covariance matrix (Banerjee et al. 2008). This key property of GPP greatly decreases the computational complexity of the model when $m\ll n_{y}$, as sampling the posterior distribution is $O\left(n_{y}m^{2}\right)$ in processing time and $O\left(n_{y}m\right)$ in memory storage (Banerjee et al. 2008). For simplicity, in this study we assigned the knot locations on a uniform grid, but other knot configurations can potentially yield improved performance (Diggle and Lophaven 2006). We apply a correction to the nonstationary marginal prior variance imposed by GPP, so that it always coincides with original GP variance (Finley et al. 2009). As far as we are aware, the most similar model that combines GPP with factor modeling was proposed by Ren and Banerjee (2013) for analysis of multivariate environmental data under the assumption of Gaussian noise.

The NNGP builds upon the conditional representation of the original GP (Datta et al. 2016). Given a specified ordering over the set of sites $S=\left[s_{1},\ldots ,s_{n_{y}}\right]$, the process $w\left(s\right)∼\text{GP}\left(0,k\left(s_{1},s_{2}\right)\right)$ over this set corresponds to multivariate Gaussian distribution $w={\left[w\left(s_{1}\right),\ldots ,w\left(s_{n_{y}}\right)\right]}^{T}∼N\left(0,K_{\mathrm{SS}}\right)$ that can be specified in the conditional manner: $w_{1}∼N\left(0,K_{11}\right)$, $\left(w_{i}|w_{j},j<i\right)∼N\left(\mu_{i},d_{i}\right)\forall i\in 2\ldots n_{y},$ where $\mu_{i}$ and $d_{i}$ are the conditional mean and variance. This leads to a factorization of the covariance matrix $K={\left(I_{n_{y}}-A\right)}^{-1}D{\left(I_{n_{y}}-A\right)}^{-T}$, where A is the strictly lower triangular matrix with elements $a_{\mathrm{ij}}$ and D is the diagonal matrix with elements $d_{i}$. The NNGP approximates the above‐defined exact conditional distribution $\left(w_{i}|w_{j},\;j<i\right)$ by conditioning only on the m preceding closest neighbors of $s_{i}$: $\left(w_{i}|w_{j},\;j\in N\left(i\right)\right)$. This results in an approximate factorization of covariance matrix $K\approx \hat{K}={\left(I-\hat{A}\right)}^{-1}\hat{D}{\left(I-\hat{A}\right)}^{-T}$, with sparse matrix $\hat{A}$; hence the precision matrix ${\hat{K}}^{-1}={\left(I-\hat{A}\right)}^{T}{\hat{D}}^{-1}\left(I-\hat{A}\right)$ is also sparse with $O\left(n_{y}m\right)$ nonzero entries. The enhanced computational efficiency of this method is achieved because of the decreased cost of sparse matrix operations compared to their dense counterparts. Recently, Taylor‐Rodriguez et al. (2018) proposed a similar blend of NNGP and latent factors to build a two‐stage probabilistic model linking together aerial LiDAR data and forest inventory observations. However, their sequential Gibbs updater for latent factors is different from our block implementation that uses sparse Cholesky as was proposed by Datta et al. (2016) and further detailed in Finley et al. (2019).

Ovaskainen et al. (2017) presented the software HMSC‐Matlab for sampling the posterior distribution of the HMSC model with a spatial structure implemented through GP with an exponential covariance function. We present an extension to this software that allows users to choose between GP, GPP, and NNGP implementations.1
https://github.com/gtikhonov/HMSC-Matlab-BigSpatial. As detailed in the Appendix S1, we devised a full‐conditional block Gibbs sampler that updates all latent factors simultaneously in a computationally efficient manner.

### Case study—plants community in Australia

We used plant data (1) to test the feasibility to apply the methods developed here to data that are large in terms of both the number of sampling sites and the number of species, and (2) to determine how their performance compares to full spatial and nonspatial model, assuming different parameters of the methods (number of knots in GPP and number of neighbors in NNGP, and number of factors in both methods). The analyzed data set involved the occurrences of 623 species recorded at 30,955 sites within the State of Victoria, Australia (Fig. 2A).

We selected four environmental covariates that were essentially uncorrelated and were considered potentially important to vegetation and plant distribution. These measure (1) climatic conditions (average maximum temperature in January), (2) water proximity (vertical distance to the nearest water body within the relevant watershed), (3) soil type (for which we used the radioelement count of thorium as a proxy—see Read et al. (2018)), and (4) solar radiation (based on the local topography). We included the squared value of each variable to allow the modeled occurrence probabilities to peak at an intermediate value of the covariate (see Appendix S1 for more details on the models).

We randomly selected 5,000 sites as validation data that were not used for model fitting. We randomly selected training data sets with $n_{y}$ = 100, 400, 1,600, 6,400 and 25,955 sites from the remaining locations, each smaller data set being included within the larger ones. To examine how the performance of the methods depended on the size of the species community, we fitted the model to subsets of $n_{s}$ = 40, 160, and 623 species. We selected these subsets uniformly from all species, sorted in terms of their prevalence, ensuring an unbiased representation of both common and rare species. We further selected the subsets iteratively so that smaller species subsets were included within the larger ones. The combination of five sample sizes and three community sizes yielded 15 data sets, which we used to compare the performance of four kinds of models, named according to what assumptions were made about latent factors: nonspatial, GP‐based, GPP‐based, and NNGP‐based. In the GPP model, we repeated all analyses with m = 16, 64, 256, and 1,024 knots, chosen as nodes of a uniform hexagonal grid covering the study area (Fig. 2A). In the NNGP model, we repeated all analysis with m = 10 and 20 neighbors. In the full GP model we restricted the analyses to $n_{y}$ ≤ 1,600, as larger data sets were not computationally feasible because of insufficient RAM. In our first analysis, we fixed the number of latent factors to $n_{f}=2$ in all models to restrain their flexibility and facilitate comparison. In our second set of analyses, we investigated the effect of number of latent factors on the predictive performance for a subset of models. We used all $n_{y}$ = 25,955 training sites, $n_{s}=40$, and $n_{s}=623$ species, GPP with 64 nodes, and NNGP with 10 neighbors, and varied the number of factors $n_{f}$ from 2 to 32.

We fitted all models with 10,000 MCMC steps, out of which we discarded the first 2,000 steps as burn in. We thinned the remaining samples by 10, resulting in 800 posterior draws. We examine the convergence of the MCMC chains by fitting the models 40 times with initial parameter values sampled from the prior distribution. We characterized the performances of the models in terms of their out‐of‐sample predictive power and computational demand. To evaluate the predictive power, we used the fitted models to predict species occurrence probabilities for the 5,000 validation sites not used for model fitting and evaluated their accuracy using Tjur’s *R* ^2^ (Tjur 2009) and deviance. To compare the computational demand, we fitted all models for the first analysis with the same software and hardware (Matlab 2017a; a desktop with Intel i5 3.00 GHz CPU and 16 GB of 1,600 MHz RAM) and evaluated the execution time required to run the model for 10,000 MCMC iterations. We additionally estimated the effective sample size of the fitted chains and evaluated the expected time required to obtain 1,000 effective posterior samples.

To illustrate ecological inference that can be derived from the modeling approaches, we used the GPP model with the largest number of knot points (m = 1,024, Fig. 2A) and the NNGP model with the largest number of neighbors (m=20), both fitted to the entire training data ($n_{y}$ = 25,955, $n_{s}=623$) with $n_{f}=2$. We visualized posterior mean correlation matrices of species associations and constructed predictive distribution maps for individual species and species richness. We further divided the study area into regions of common composition profile, performing clustering with a 5 × 5 self‐organizing map that seeks to assign similar species composition profiles to nearby clusters (Kohonen 1982).

## Results

### Comparison of predictive performance and execution times

**Figure 1:**
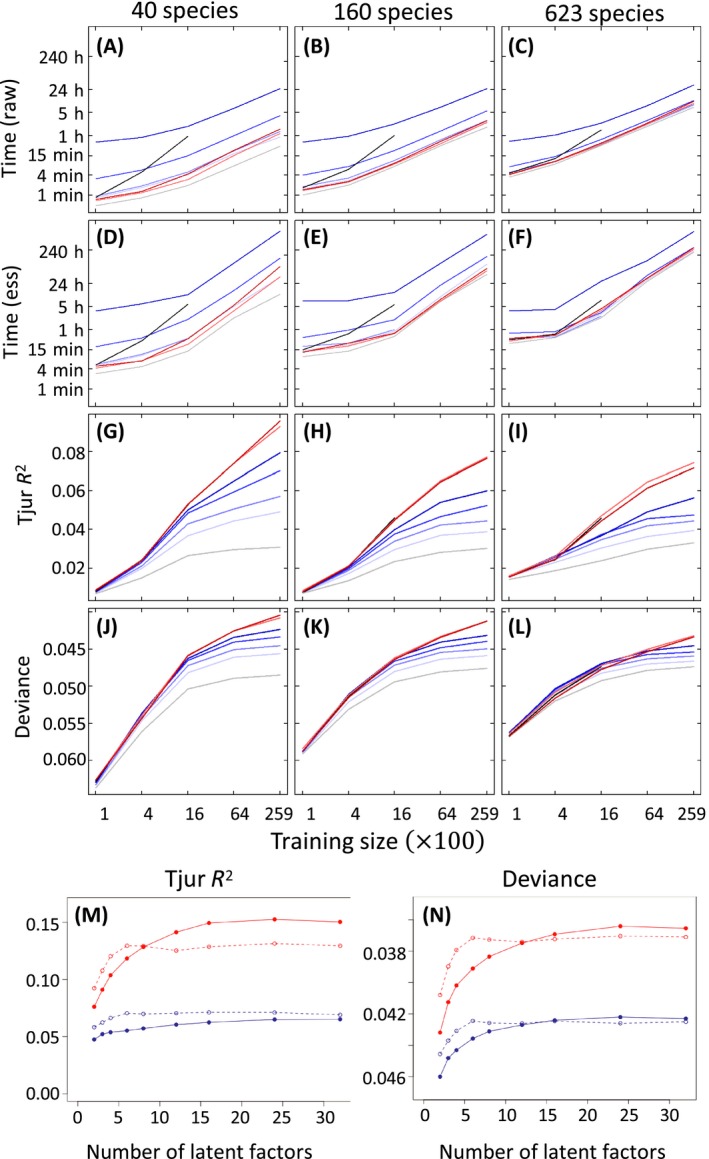
Comparison of nonspatial, full Gaussian process (GP), Gaussian predictive process (GPP), and nearest‐neighbor Gaussian process (NNGP) models. Panels (A)–(C) show time elapsed for model fitting to small ($n_{s}=40$), medium ($n_{s}=160$), and large ($n_{s}=623$) species communities with $n_{f}=2$ using a hierarchical model of species communities (HMSC) Gibbs sampler with 10,000 Markov chain Monte Carlo (MCMC) iterations. Panels (D)–(F) depict the same results adjusted for the autocorrelation in the samples, showing the time required to obtain 1,000 effectively independent samples from the posterior. Panels (G)–(I) show predictive performance measured in terms of Tjur *R* ^2^ for models fitted, and panels (J)–(L) in terms of deviance. The colors indicate nonspatial models (gray), GP models (black), GPP models with 16, 64, 265, and 1,024 knots (gradation of blue from light to deep), the NNGP models with 10 and 20 neighbors (light and dark red). Note that because of very similar results, red and black lines often overlap. Panels (M) and (N) depict the predictive performance results with respect to number of factors. Dashed lines depict cases with $n_{s}=40$ species and solid lines cases with $n_{s}=623$ species; blue lines correspond to GPP with 64 knots and red lines correspond to NNGP with 10 neighbors.

Predictive performance generally increased with model complexity, so that the nonspatial model performed the worst, and the performance of the predictive process improved with the number of knots (Fig. 1). Even a very coarse approximation of spatial structure with only 16 knots provided a substantial gain in the predictive performance, as compared to the nonspatial model. The performances of the GP and both NNGP models were essentially equal and outperformed the GPP model when the number of knots was lower than number of training points. Our results with the number of factors fixed to $n_{f}=2$ suggest that predictive performance reduces with increasing size of the community (Fig. 1G–L). This behavior is at least partially due to the fact that predictive performance increases with the number factors especially for the case of many species (Fig. 1M, N), i.e., successful modeling of many species calls for many factors.

**Figure 2:**
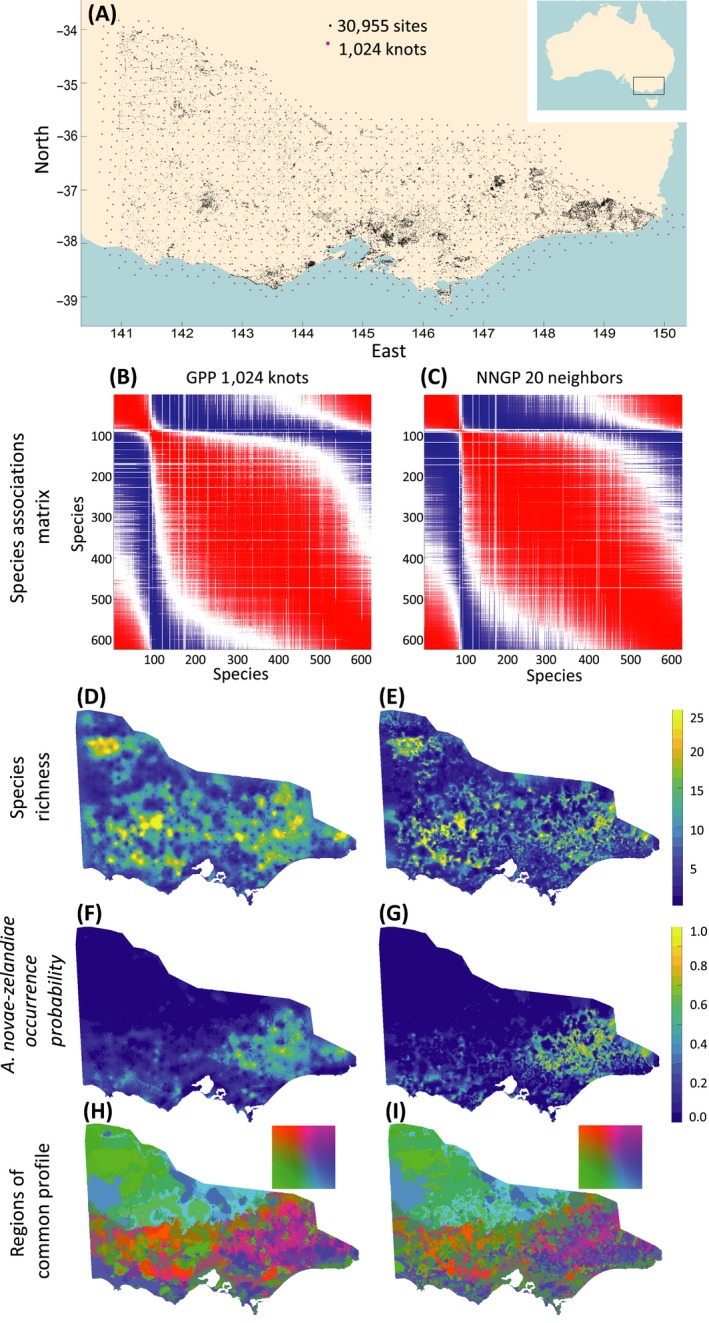
Ecological inference with Gaussian predictive process (GPP) and nearest‐neighbor Gaussian process (NNGP) models fitted to the full training data set. Panel (A) shows the spatial locations of observed sites (black), and 1,024 knots used in the biggest GPP model (magenta). Panels (B) and (C) show species association patterns, with red (respectively, blue) depicting species pairs that co‐occur more often (respectively, less often) based on the latent factor part of the hierarchical model of species communities (HMSC) model, and white color stands for the species pairs for which association sign was not credibly estimated at 95% threshold. Species ordering is the same in both panels and selected for enhanced visual clarity of association structure. Panels (D) and (E) visualize predicted spatial distribution of species richness, (F) and (G)—predicted occurrence probability of *Acaena novae‐zelandiae*; (H) and (I)—predicted regions of common profile, with nodes of 5 × 5 self‐organizing map mapped to YUV color space.

The computational times needed for a single Gibbs update step were consistent with theoretical expectations (Appendix S1): the computational time increased approximately linearly with sample size in nonspatial and GPP models, slightly faster in NNGP, and cubically in the full GP. The effective sample size substantially decreased with increased number of training sites, which is a known deficiency of the classic probit data augmentation scheme (Duan et al. 2017) applied in HMSC. Thus, the computational time needed for obtaining a given effective number of posterior samples increases steeper with number of sample size than the time per MCMC iteration (Fig. 1A–F). The comparison of 40 independent MCMC chains showed that obtaining satisfactory mixing in the spatial models with large numbers of sampling units and species is challenging (Appendix S1). This suggests that the performance of the spatial models reported in Fig. 1 may be suboptimal, even if the models present a clear improvement over the nonspatial model.

### Ecological inference with GPP and NNGP

The GPP and the NNGP provided essentially identical estimates of species association matrices, revealing numerous positive and negative residual associations (Fig. 2B, C). However, these models substantially differed in their spatial predictions (Fig. 2D–I). The NNGP model predicted more fine‐scaled patterns and exhibited discontinuities, especially in areas distant from the training sites. The GPP model predicted smoother patterns that in some regions vaguely resembled the structure of the grid of knots used.

## Discussion

In this paper, we have transferred methods from spatial statistics (Banerjee et al. 2008, Datta et al. 2016) to enable statistical modeling of species communities with big spatial data. The HMSC model augmented with a GPP or a NNGP displayed much better scaling of computational complexity than the originally proposed spatial HMSC, and much better performance than the nonspatial HMSC. Our results indicate that the NNGP‐augmented HMSC performs the best in terms of the trade‐off between computational time and predictive performance, which mirrors similar findings for univariate models (Datta et al. 2016). However, the superiority of NNGP over GPP may have been favored by some case‐specific factors. First, the spatial range of the latent factors in our study was estimated to be rather small, making only nearby locations effectively nonindependent. Second, the spatial distribution of sampling sites in our data was spatially uneven, with multiple sites often closely proximal to each other. Both these features naturally suit the NNGP approximation’s assumptions but require GPP with a uniform distribution of knots to feature a very high number of knots to approximate the original GP closely. We further note that the NNGP approach leads to rather discontinuous spatial predictions. If this is considered inconsistent with the studied ecological phenomena, an ecologist may wish to apply the GPP, for example, for making predictive maps even if it performs worse in cross validation. We also note that, in addition to the considered GPP and NNGP, there exist other prominent spatial statistical methods (Heaton et al. 2018) that could prove useful for spatial JSDMs in the future.

Our results indicate that obtaining satisfactory MCMC convergence is challenging for large data. As the challenge is present also in nonspatial models, the deficiency of the traditional data augmentation approach for probit model is likely to be the main source of the problem (Duan et al. 2017). On top of this, Gibbs MCMC convergence can be especially difficult in the spatial models because of conditional interdependencies of model components, as shown by Finley et al. (2019) for univariate NNGP with Gaussian noise. One possible solution might build on the approximate inference techniques for GPP‐like models with non‐Gaussian responses (Hensman et al. 2015), but the applicability of similar approach to NNGP is yet to be explored.

The methodological advances presented in this work facilitate the efficient use of rapidly accumulating high‐resolution large‐scale ecological data sets toward explaining and predicting how ecological communities are structured and how they respond to ongoing global change. Our implementations of GPP‐ and NNGP‐based latent factors to HMSC also allow researchers to integrate such analyses with information on species traits and phylogenetic relationships, providing the potential to address a large number of fundamental and applied questions in community ecology (Ovaskainen et al. 2017). As we have briefly illustrated with our case study on Australian plants, the methods developed here open a great array of possibilities for ecologists working on problems related to fundamental or applied community ecology, conservation biology, and macroecology. Most importantly, it is now possible to use spatially extensive data to examine how species occurrences and co‐occurrences are associated with environmental variation, how species traits and phylogenies influence such variation, and to generate and validate predictive maps at the levels of single species and community characteristics.
