# Supplementary material for: Computationally efficient joint species distribution modeling of big spatial data
Source: Ecology. 2019 Dec 20;101(2):e02929. doi: 10.1002/ecy.2929 (PMC7027487; doi:10.1002/ecy.2929)
Supplement: Supplementary file 1 [file ECY-101-e02929-s001.pdf]

Supporting Information. Gleb Tikhonov, Li Duan, Nerea Abrego, Graeme Newell, Matt White, David Dunson, and Otso Ovaskainen. 2019. Computationally efficient joint species distribution modeling of big spatial data. *Ecology.*

Appendix S1.

Contents

HMSC specification in matrix notation 2

- Indices 2
- Data objects in the model 2
- Model parameters 2
- Matrix-vector notation 2
- Distributions 2
- The Hierarchical Model of Species Communities 3
- Role of traits and phylogeny 3
- Priors 3
- Selecting the number of latent factors 4

Approximate Gaussian process priors for latent factors for big spatial data 4

- Gaussian predictive process 4
- Nearest Neighbor Gaussian process 5

Gibbs MCMC sampling algorithm 6

- Gaussian predictive process 6
  - Full-conditional updater for H 6
  - Full-conditional updater for α 7
- Nearest Neighbor Gaussian process 7
  - Full-conditional updater for H 7
  - Full-conditional updater for α 8
- Lemma 1: on full conditional updater of H for latent factors with GPP prior 8

Posterior predictive distribution 10

Details on Australian plants case study 10

Details on model convergence 11

References 17

HMSC specification in matrix notation

The HMSC specification, provided in Materials & Methods section of the main text also could be elegantly and rigorously formally written in matrix form using following notation:

Indices

We index by $i = 1 \dots n_y$ the sites, where the observations were made, by $j = 1 \dots n_s$ the species, by $k = 1 \dots n_c$ the available covariates, by $l = 1 \dots n_t$ the available traits, by $h = 1 \dots n_f$ the latent factors at the level of sites, and by $d = 1 \dots n_d$ the dimensions of the space that describes sites' locations.

Data objects in the model

- The $n_y \times n_s$ matrix Y of the recorded species abundances/occurrences y_{ij}
- The $n_y \times n_c$ matrix X of the covariates x_{ik}
- The $n_s \times n_t$ matrix T of the species traits t_{jl}
- The $n_s \times n_s$ symmetric positive definite matrix C of the species phylogenetic similarities
- The $n_y \times n_d$ matrix S of the sites' coordinates s_{id}

Model parameters

- The $n_y \times n_s$ matrix L of latent variables l_{ij} standing for location parameters of the data distribution
- The $n_c \times n_s$ matrix B of the β_{kj} of the species responses to the covariates
- The $n_c \times n_s$ matrix M of the μ_{kj} of the trait-expected species responses to the covariates
- The $n_c \times n_t$ matrix Γ of the γ_{kl} the impacts of trait values on the expected species response to the covariates
- The $n_c \times n_c$ matrix V standing for the covariance of responses to covariates across species that could not be attributed to available traits.
- Scalar ρ standing for the strength of the impact of phytogenic similarity to similarity in responses to covariates.
- The $n_f \times n_s$ matrix Λ is the matrix of latent factor loadings λ_{hj}
- The $n_y \times n_f$ matrix H of the latent factors η_{ih}
- The $n_f \times 1$ vector α of the spatial ranges of latent factors.
- The $n_y \times n_s$ matrix Z of latent liabilities $z_{ij} = l_{ij} + \varepsilon_{ij}$ used for implementation of various types of observational data through data augmentation. For theoretical ground see e.g. Albert and Chib (1993) or Zhou et al. (2012), for HMSC-contexed usage see e.g. Ovaskainen et al (2016a)
- The $n_s \times n_s$ diagonal matrix Σ of residual variances, with diagonal elements σ_j^2

Matrix-vector notation

We denote by $\mathrm{vec}(\cdot)$ the operator which stacks consecutive columns of a matrix on top of each other. We denote by small letters in **bold** font the vectors that are obtained by applying $\mathrm{vec}(\cdot)$ to corresponding matrices, so that e.g. $\boldsymbol{\gamma} = \mathrm{vec}(\Gamma)$, $\boldsymbol{\beta} = \mathrm{vec}(\mathrm{B})$, $\boldsymbol{\lambda} = \mathrm{vec}(\Lambda)$, $\boldsymbol{\eta} = \mathrm{vec}(\mathrm{H})$, $\boldsymbol{l} = \mathrm{vec}(\mathrm{L})$, $\boldsymbol{z} = \mathrm{vec}(\mathrm{Z})$. A star in the upperscript indicates that the transpose was applied first to the matrix, so e.g. $\boldsymbol{z}^* = \mathrm{vec}(\mathrm{Z}^T)$ and $\boldsymbol{\eta}^* = \mathrm{vec}(\mathrm{H}^T)$. We denote by $0_{n\times m}$ the $n \times m$ matrix of ones, by I_n the $n \times n$ identity matrix, by $\mathrm{tr}(\mathrm{A})$ the trace of the matrix A, by $\otimes$ the Kronecker product, and by $\circ$ the Hadamard product (the entry-wise product).

Distributions

- We denote by $N(\boldsymbol{\mu}, \Sigma)$ the multivariate normal (Gaussain) distribution with mean $\boldsymbol{\mu}$ and covariance matrix Σ.
- We denote by $\mathrm{W}(\mathrm{V}, \upsilon)$ the Wishart distribution with scale matrix V and degrees of freedom parameter υ, and by $\mathrm{W}^{-1}(\mathrm{V}, \upsilon)$ the inverse Wishart distribution with scale matrix V and degrees of freedom parameter υ. Thus if $\mathrm{V} \sim \mathrm{W}^{-1}(\mathrm{V}_0, \upsilon)$, then $\mathrm{V}^{-1} \sim \mathrm{W}(\mathrm{V}_0^{-1}, \upsilon)$.

- We denote by $\Gamma(a, b)$ the Gamma distribution with shape a and rate b, which parametrization is common to Bayesian statistics, so that the distribution's mean is a/b.

The Hierarchical Model of Species Communities

We follow the generalized linear modelling paradigm and model that the observations for the j-th species with a statistical distribution D_j and link function g_j that are compatible with the type of observed data for this species. Then

$$E(y_{ij}) = g_j^{-1}(l_{ij}), \quad Var(y_{ij}) = Var_{D_j}(l_{ij}, \sigma_j), \quad \mathrm{L} = \mathrm{XB} + \mathrm{H}\Lambda,$$

$$\boldsymbol{\beta} \sim N(\boldsymbol{\mu}, \Theta), \qquad \boldsymbol{\mu} = \mathrm{vec}(\Gamma\mathrm{T}^T) = (\mathrm{T} \otimes \mathrm{I}_{n_c})\boldsymbol{\gamma}, \qquad \Theta = [\rho\mathrm{C} + (1-\rho)\mathrm{I}_{n_s}] \otimes \mathrm{V}$$

We would like to mention, that while in this study we consider only one level of random factors in the model, which corresponds to level of single observational sites, the proposed model is trivially generalizable to more complex sampling designs with several hierarchical or crossed levels, see e.g. Ovaskainen et al. (2016a).

Role of traits and phylogeny

To improve the performance of the model with sparse data or rare species, as well as to exploit potentially available information on species-specific traits and phylogenetic relationships, we follow Ovaskainen et al. (2017) and impose a multivariate Gaussian conditional prior for the regression coefficients as $\boldsymbol{\beta} = [\beta_{1\cdot}, \dots, \beta_{n_c\cdot}]^T \sim N(\mathrm{vec}(\mathrm{M}^T), \Theta)$. The matrix $\mathrm{M} \in \mathbb{R}^{n_c \times n_s}$ consists of the elements μ_{kj} that describe the expected response of species j to covariate k. The expected response of species j to covariate k is modeled based on this species' traits as $\mu_{kj} = \sum_{l=1}^{n_t} \gamma_{kl} t_{jl}$, where t_{jl} is the value of trait l for species j (with $t_{j1} = 1$ modeling the intercept), and the parameter γ_{kl} measures the effect of trait l on the expected response to covariate k. Matrix Θ models the variation of responses among individual species around the trait-based expectation as $\Theta = [\rho\mathrm{C} + (1-\rho)\mathrm{I}_{n_s}] \otimes \mathrm{V}$, where $\otimes$ denotes Kronecker's product, the positive-definite matrix $\mathrm{V} \in \mathbb{R}^{n_c \times n_c}$ models the dependency between responses to different covariates, the parameter $0 \leq \rho \leq 1$ determines the impact of phylogenetic relationships on species responses to the covariates, and the matrix $\mathrm{C} \in \mathbb{R}^{n_s \times n_s}$ is a phylogenetic correlation matrix, which is assumed to be known prior to the analysis. The model can be applied without trait data by including the intercept as the only species trait, and it can be applied without phylogenetic data by fixing $\rho = 0$.

Priors

Here we list the families of the priors that we assign, and which are essential for our sampling algorithm. We also provide the "default" values for those, which correspond to weakly informative prior that is recommended for practical use in case when no or very few additional information is available a priori (applied with normalized input data).

- The prior for V is the inverse-Wishart prior with parameters V_0 and f_0, $\mathrm{V} \sim \mathrm{W}^{-1}(\mathrm{V}_0, f_0)$. The prior parameters are V_0 ($n_c \times n_c$ covariance matrix) and f_0 (scalar). Their default values are $\mathrm{V}_0 = \mathrm{I}_{n_c}$ and $f_0 = n_c + 1$.
- The prior for γ is $\gamma \sim N(m_\Gamma, \mathrm{U}_\Gamma)$. The prior's hyperparameters are m_Γ (vector of length $n_c n_t$) and U_Γ (covariance matrix of dimension $n_c n_t \times n_c n_t$). Their default values $m_\Gamma = 0_{n_c n_t \times 1}$ and $\mathrm{U}_\Gamma = \mathrm{I}_{n_c n_t}$.
- If phylogeny is included, the prior for $0 \leq \rho \leq 1$ is a discrete prior that approximates spike-and-slab, with values ρ_g and weights w_g. The default values are $\rho_g = (g-1)/100$ and $w_1 = 0.5$, $w_g = 1/200$ for $g = 2, \dots, 101$. If phylogeny is not included, this parameter is fixed to $\rho = 0$.
- The prior for those diagonal elements of Σ that are not fixed due to the selected data distribution D_j is $\sigma_j^{-2} \sim \mathrm{Ga}(a_j, b_j)$. The prior parameters are the scalars a_j and b_j. Their default values are $a_j = 1$ and $b_j = 0.3$.
- For $\Lambda_k \Lambda_k^{\mathrm{T}}$, we assume the multiplicative gamma process shrinkage prior suggested by Bhattacharya and Dunson (2011), in which the degree of shrinkage increases with the factor number. Thus,

$$\lambda_{jh} | \phi_{jh}, \tau_h \sim N(0, 1/(\tau_h \phi_{jh})), \quad \phi_{jh} \sim \mathrm{Ga}\left(\frac{v}{2}, \frac{v}{2}\right), \quad \tau_h = \prod_{l=1}^{h} \delta_l,$$

$$\delta_1 \sim \mathrm{Ga}(a_1, b_1), \delta_l \sim \mathrm{Ga}(a_2, b_2) \text{ for } l \geq 2.$$

- The authors of this method proposed that the parameter υ is fixed to $\upsilon = 3$ and the parameters b_1 and b_2 are fixed to $b_1 = b_2 = 1$. The parameters a_1 and a_2 are selected by the user to define the level of shrinkage , with a_1 tuning the basic level and $a_2 > 1$ the increase in shrinkage with increasing number of the factor. The default values are $a_1 = a_2 = 5$. In case of many species with sparse data, it may be useful to increase these parameters to increase shrinkage (and thus decrease the estimated number of latent factors), although the original study claims that the method is quite robust to the choice of these hyperparameters.
- If the latent factors are not spatially structured, the prior for H is $\boldsymbol{\eta} \sim N\left(0, \mathrm{I}_{n_f} \otimes \mathrm{I}_{n_y}\right)$. If the latent factors are spatially structured, the prior for H is $\boldsymbol{\eta} \sim N(0, \mathrm{K_H})$, where $\mathrm{K_H}$ is the block-diagonal matrix $\mathrm{K_H} = \mathrm{diag}(\mathrm{K}^1, \dots, \mathrm{K}^{n_f})$, where $\mathrm{K}^h_{ii'} = f^h\left(\|x_i - x_{i'}\|_2, \alpha^h\right)$. Here $f^h\left(d, \alpha^h\right)$ is some isotropic stationary covariance function with $f^h\left(0, \alpha^h\right) = 1$, and α^h is its spatial range parameter. The prior for α^h is a discrete prior that approximates spike-and-slab, with values α_g and weights w_g, $g = 1 \dots n_{\alpha_g}$. The default values are the exponential covariance function $f^h\left(d, \alpha^h\right) = \exp\left(-d/\alpha^h\right)$, $\alpha_g = d_{max}(g-1)/100$ and $w_1 = 0.5$, $w_g = 1/200$ for $g = 2, \dots, 101$, where d_{max} is the largest distance between the sites.

Selecting the number of latent factors

Determining the appropriate number of latent factors is fundamental for the HMSC model specified above. While in certain cases, this number can be obtained based on an informed expert opinion guess, generally such information is unavailable before the analysis. Some previous works have suggested methods for a proper Bayesian treatment of n_f, and estimating it during the MCMC sampling (Lopes and West 2004). However, such scheme requires changing the domain of the parameter space during sampling and the proposed reversible-jump MCMC does not scale well. Instead, Bhattacharya and Dunson (2011) devised a formulation of infinite factor model, where the number of latent factors is assumed to be infinite, but the latent loadings of higher factors are shrink by assigning multiplicative Gamma process prior. In practice, the authors proposed to use adaptive tuning of n_f during the warm-up phase of MCMC scheme, based on discarding the latent factors, which latent loadings do not exceed certain pre-defined threshold. This method was implemented in the HMSC implementation presented in Ovaskainen et al. (2017) and consequently is used in this work.

However, our experience with HMSC model indicates that with insufficient adaptation period or too severe shrinkage the estimated number of latent factors can be suboptimal, while with too mild shrinkage, the estimated number of factors can be unnecessarily high. The later generally does not affect the quality of the model fit due to originally infinite number of factors assumed, but is undesirable in spatial models, since it dramatically increases the computational load. An even more robust, although numerically very costly scheme to estimate the proper number of latent factors is to iteratively run several instances of HMSC model in cross-validation manner, while varying the number of latent factors. Then, the model with best cross-validation predictive performance is likely to contain the number of latent factors that is close to the truly optimal (may depend on the of CV fold splitting strategy).

Approximate Gaussian process priors for latent factors for big spatial data

Gaussian predictive process

The Gaussian predictive process (GPP) denoted by $\widetilde{w}(s)$, is constructed from the values of the original GP $w(s)$ defined at m 'knot' locations $S^* = \{s_1^*, \dots, s_m^*\}$. Therefore, the value of the GPP at any site s_0 is given by $\widetilde{w}(s_0) = E(w(s_0)|w^*) = K_{s_0 S^*} K_{S^* S^*}^{-1} w^*$, where $w^* = [w(s_1^*), \dots, w(s_m^*)]^T$ denotes the vector of the original GP values at the knot locations S^*, $K_{S^* S^*} = \left[k\left(s_{i_1}^*, s_{i_2}^*\right)\right]_{i_2=1\dots m}^{i_1=1\dots m}$ and $K_{s_0 S^*} = [k(s_0, s_1^*), \dots, k(s_0, s_m^*)]$. With this definition, it follows that $\widetilde{w}$ is itself a GP: $\widetilde{w}(s) \sim \mathrm{GP}\left(0, \tilde{k}(s_1, s_2)\right)$, where the covariance function $\tilde{k}(s_1, s_2) = K_{s_1 S^*} K_{S^* S^*}^{-1} K_{s_2 S^*}^T$ is non-stationary but factorizable

(Banerjee et al. 2008). As mentioned in the main text, our interpretation of the covariance matrix Ω assumes that the marginal prior distributions of the latent factors is standard normal. However, the GPP fails to fulfill that the marginal distribution of latent factors is standard normal since its marginal variance generally decreases with increasing distance from the knot set S^*. To circumvent this misbehavior, we followed Finley et al. (2009) and applied a correction to the marginal prior variance of the GPP, so that it always equals that of the original GP: $\hat{k}(s_1, s_2) = K_{s_1 S^*} K_{S^* S^*}^{-1} K_{s_2 S^*}^T + \delta(s_1 = s_2)\left(k(s_1, s_2) - K_{s_1 S^*} K_{S^* S^*}^{-1} K_{s_2 S^*}^T\right)$.

Hence, the prior for H is $\boldsymbol{\eta} \sim N\left(0, \widehat{\mathrm{K}}_{\mathrm{H}}\right)$, where $\widehat{\mathrm{K}}_{\mathrm{H}}$ is the block-diagonal matrix $\widehat{\mathrm{K}}_{\mathrm{H}} = \mathrm{diag}\left(\widehat{\mathrm{K}}^1, \ldots, \widehat{\mathrm{K}}^{n_f}\right)$, and $\widehat{K}_{ii'}^h = \hat{k}^h(s_i, s_{i'})$. Alternatively, $\widehat{\mathrm{K}}^h = K_{SS^*}^h \left(K_{S^*S^*}^h\right)^{-1} \left(K_{SS^*}^h\right)^T + D^h$, where D^h is diagonal matrix that corrects for the marginal variances with elements $d_{ii}^h = 1 - K_{s_i S^*}^h \left(K_{S^*S^*}^h\right)^{-1} \left(K_{s_i S^*}^h\right)^T$. The elements d_{ii}^h are guaranteed to be non-negative as they are variances in conditional Gaussian distributions of $w(s_i)$, conditional on $[w(s_1^*), \ldots, w(s_m^*)\ ]$, and therefore, $\widehat{\mathrm{K}}^h$ is a valid correlation matrix.

As far as we are aware, the most similar model to GPP-augmented HMSC was proposed by Ren and Banerjee (2013), where the authors also coupled GPP with factor modelling for analysis of multivariate environmental data under the assumption of Gaussian noise.

Nearest Neighbor Gaussian process

Nearest Neighbor Gaussian Process (NNGP) builds upon a special sparse approximation of the GP precision matrix that is related to the conditional representation of the original GP (Datta et al. 2016b). Given a specified ordering over the set of sites $S = \left[s_1, \ldots, s_{n_y}\right]$ the process $w(s) \sim \mathrm{GP}\left(0, k(s_1, s_2)\right)$ over this set corresponds to multivariate Gaussian distribution $\boldsymbol{w} = \left[w_1, \ldots, w_{n_y}\right]^T = \left[w(s_1), \ldots, w\left(s_{n_y}\right)\right]^T \sim N(0, K_{SS})$ that can be specified in conditional manner:

$$w_1 \sim N(0, K_{11}), \qquad (w_i | w_j, j < i) \sim N(\mu_i, d_i)\ \forall i \in 2 \ldots n_y$$

$$\mu_i = \sum_{j=1}^{i-1} a_{ij} w_j\,, \qquad \left[a_{i,1}, \ldots, a_{i,i-1}\right]^T = \left(\left[K_{j_1 j_2}\right]_{j_2=1\ldots i-1}^{j_1=1\ldots i-1}\right)^{-1} \left[K_{1i}, \ldots, K_{i-1,i}\right]^T,$$

$$d_i = K_{ii} - \left[K_{1i}, \ldots, K_{i-1,i}\right]\left[a_{i,1}, \ldots, a_{i,i-1}\right]^T$$

This leads to a factorization of the covariance matrix $K = \left(\mathrm{I}_{n_y} - \mathrm{A}\right)^{-1} \mathrm{D} \left(\mathrm{I}_{n_y} - \mathrm{A}\right)^{-T}$, where A is the strictly lower triangular matrix with elements a_{ij} and D is the diagonal matrix with elements d_i. The Nearest Neighbor approach approximates the conditional distribution $(w_i | w_j, j < i) \sim N(\mu_i, d_i)$ by conditioning only on the m preceding closest neighbors of s_i: $(w_i | w_j, j < i) \approx \left(w_i | w_j, j \in N_m(i)\right)$, where $N_m(i) = \left[n_1^i, \ldots, n_{\widetilde{m}}^i\right]$ is the subset of $\{1, \ldots, i-1\ \}$ of size $\widetilde{m} = \min(m, i-1)$ that contains the indices of at most m closest neighbors of s_i. This results in the following adjusted formulas:

$$w_1 \sim N(0, K_{11}), \qquad \left(w_i | w_j, j \in N(i)\right) \sim N\left(\tilde{\mu}_i, \tilde{d}_i\right)\ \forall i \in 2 \ldots n_y$$

$$\tilde{\mu}_i = \sum_{j \in N_m(i)} a_{ij} w_j\,, \qquad \left[a_{i,n_1^i}, \ldots, a_{i,n_{\widetilde{m}}^i}\right]^T = \left(\left[K_{j_1 j_2}\right]_{j_2=n_1^i,\ldots,n_{\widetilde{m}}^i}^{j_1=n_1^i,\ldots,n_{\widetilde{m}}^i}\right)^{-1} \left[K_{n_1^i,i}, \ldots, K_{n_{\widetilde{m}}^i,i}\right]^T,$$

$$d_i = K_{ii} - \left[K_{n_1^i,i}, \ldots, K_{n_{\widetilde{m}}^i,i}\right]\left[a_{i,n_1^i}, \ldots, a_{i,n_{\widetilde{m}}^i}\right]^T$$

approximate factorization of covariance matrix $\mathrm{K} \approx \widehat{\mathrm{K}} = \left(\mathrm{I} - \widehat{\mathrm{A}}\right)^{-1} \widehat{\mathrm{D}} \left(\mathrm{I} - \widehat{\mathrm{A}}\right)^{-T}$ with sparse matrix $\widehat{\mathrm{A}}$, which non-zero elements are obtained via the expressions above. Hence the precision matrix $\widehat{\mathrm{K}}^{-1} = \left(\mathrm{I} - \widehat{\mathrm{A}}\right)^T \widehat{\mathrm{D}}^{-1} \left(\mathrm{I} - \widehat{\mathrm{A}}\right)$ is also sparse with $O\left(n_y m^2\right)$ non-zero entries. Another crucial property of this matrix is that all its non-zero elements tend to be close to the diagonal (the exact measure of how far the non-zero elements could be away from the diagonal depends on the coordinates of the sites and selected ordering; the practical advice is that the ordering should be selected to minimize it in

order to enhance performance). This imposes that the Cholesky decomposition $LL^T = \widehat{\mathrm{K}}^{-1} + \overline{D}$, where $\overline{D}$ is a diagonal matrix with non-negative elements is also sparse. The enhanced computational efficiency of the NNGP method is achieved due to the decreased cost of sparse matrix operations compared to their dense counterparts. A detailed review of how the sparsity of NNGP can be harnessed for numerical speed-up of Bayesian inference we is given in Finley et al. (2019).

Therefore, the prior for H is $\boldsymbol{\eta} \sim N(0, \widehat{\mathrm{K}}_{\mathrm{H}})$, where $\widehat{\mathrm{K}}_{\mathrm{H}}$ is the block-diagonal matrix $\widehat{\mathrm{K}}_{\mathrm{H}} = \mathrm{diag}(\widehat{\mathrm{K}}^1, \dots, \widehat{\mathrm{K}}^{n_f})$, and $\widehat{\mathrm{K}}^h = (\mathrm{I} - \widehat{\mathrm{A}}^h)^{-1} \widehat{\mathrm{D}}^h (\mathrm{I} - \widehat{\mathrm{A}}^h)^{-T}$. Alternatively, $\widehat{\mathrm{K}}_{\mathrm{H}}^{-1} = \mathrm{diag}\left((\widehat{\mathrm{K}}^1)^{-1}, \dots, (\widehat{\mathrm{K}}^{n_f})^{-1}\right)$, and $(\widehat{\mathrm{K}}^h)^{-1} = (\mathrm{I} - \widehat{\mathrm{A}}^h)^{-1} \widehat{\mathrm{D}}^h (\mathrm{I} - \widehat{\mathrm{A}}^h)^{-T}$.

Recently, Taylor-Rodriguez et al. (2018) proposed a similar blend of NNGP and latent factors to build a 2-stage probabilistic model linking together areal LiDAR data and forest inventory observations. However, the *sequential* Gibbs updater for latent factors implemented in that work, is principally different from our *block* implementation that follows the original note on using sparse Cholesky by Datta et al. (2016a).

One practical challenge related to NNGP is that the approximation is non-invariant w.r.t. the selected ordering of the set of locations. Vecchia (1992) and Stein et al. (2004) asserted that similar conditional approximations are non-sensitive to the ordering. (Datta et al. 2016b) conducted a numerical experiment that demonstrated that results are practically invariant to the ordering choice in terms of root mean square predictive errors this choice when the ordering is selected along any direction in the spatial coordinate space. However, the maximum-minimum distance ordering, recently proposed by Guinness (2018) resulted in substantially lower Kullback–Leibler (KL) divergence of approximation, compared to the geographical gradient ordering. Further, in multivariate case there is a possibility that the minimum posterior KL divergence between the original GP-based HMSC model and NNGP-based approximation could be achieved using different orderings for different latent factors. Therefore, to keep the focus on practical multivariate ecological spatial data analysis specifics of our study, we left the ordering choice comparisons to other studies and in the presented experiments always ordered the sites according to their longitude from West to East.

Gibbs MCMC sampling algorithm

We extended Gibbs posterior sampling algorithm for standard HMSC's parameters for the case when latent factors η_{ih} are assigned with Gaussian predictive process or Nearest Neighbor Gaussian process approximations of their original full Gaussian process prior. We implemented the extended sampling algorithm in the Matlab version of the HMSC package (Ovaskainen et al. 2017). Here we only present those steps from the overall sampling scheme that differ from previously published works (Bhattacharya and Dunson 2011, Ovaskainen et al. 2016a, Ovaskainen et al. 2016b, Ovaskainen et al. 2017, Tikhonov et al. 2017), namely the full-conditional updaters for H and α.

Gaussian predictive process

Full-conditional updater for H

If the latent factors are assigned GPP prior, then the full-conditional distribution for $\boldsymbol{\eta}$ follows:

$$(\boldsymbol{\eta}| -) \sim N(\boldsymbol{\mu}_\eta, \mathrm{U}_\eta), \qquad \mathrm{U}_\eta^{-1} = \widehat{\mathrm{K}}_{\mathrm{H}}^{-1} + \Lambda^T \Sigma^{-1} \Lambda \otimes I_{n_y}, \qquad \boldsymbol{\mu}_\eta = \mathrm{U}_\eta \mathrm{vec}(Z \Sigma^{-1} \Lambda)$$

However, the direct calculation does not bring any computational advantage compared to using latent factors assigned with GP prior. Instead, $\boldsymbol{\eta}$ could be drawn from the following expressions (see Lemma 1 for details on computations)

$$\boldsymbol{\eta} = A^{-1} \mathrm{vec}(Z\Sigma^{-1}\Lambda) + (A^{-1}D^{-1}W_{12}L_{F^{-1}})\left((A^{-1}D^{-1}W_{12}L_{F^{-1}})^T \mathrm{vec}(Z\Sigma^{-1}\Lambda)\right) + L_{A^{-1}}\boldsymbol{z}_1 + (A^{-1}D^{-1}W_{12}L_{F^{-1}})\boldsymbol{z}_2$$

$$W_{12} = diag\left(K_{SS^*}^1, \dots, K_{SS^*}^{n_f}\right), \qquad W_{21} = W_{12}^T, \qquad W_{22} = diag\left(K_{S^*S^*}^1, \dots, K_{S^*S^*}^{n_f}\right), \qquad D = diag(D^1, \dots, D^{n_f})$$

$$A = \Lambda^T \Sigma^{-1} \Lambda \otimes I_{n_y} + D^{-1}, \qquad F = M - W_{21} D^{-1} A^{-1} D^{-1} W_{12}, \qquad L_{A^{-1}} L_{A^{-1}}^T = A^{-1}, \qquad L_{F^{-1}} L_{F^{-1}}^T = F^{-1}$$

$$\boldsymbol{z}_1 \sim N\left(0_{n_y n_f \times 1}, I_{n_y n_f}\right), \qquad \boldsymbol{z}_2 \sim N\left(0_{n_k n_f \times 1}, I_{n_k n_f}\right)$$

Full-conditional updater for α

Conditioning on H, spatial range parameters α_h are independent for $h = 1 \dots n_f$ and can be sampled one by one from the prior values α_g proportional to their conditional posterior probabilities. We follow the sampling scheme presented in Ovaskainen et al. (2016b), but exploit the special structure of GPP-induced covariance matrices $\widehat{\mathrm{K}}^h$.

For each latent factor this scheme requires n_{α_g} calculations of the quadratic form $\eta_{\cdot h}^T\left(\widehat{\mathrm{K}}^h\right)^{-1}\eta_{\cdot h}$, and additionally n_{α_g} calculations of $\left|\widehat{\mathrm{K}}^h\right|$ that are shared among the latent factors.

$$\begin{aligned}
\eta_{\cdot h}^T\left(\widehat{\mathrm{K}}^h\right)^{-1}\eta_{\cdot h} &= \eta_{\cdot h}^T\left(K_{SS^*}^h\left(K_{S^*S^*}^h\right)^{-1}\left(K_{SS^*}^h\right)^T + D^h\right)^{-1}\eta_{\cdot h} \\
&= \eta_{\cdot h}^T\left(\left(D^h\right)^{-1} - \left(D^h\right)^{-1}K_{SS^*}^h\left(K_{S^*S^*}^h + \left(K_{SS^*}^h\right)^T\left(D^h\right)^{-1}K_{SS^*}^h\right)^{-1}\left(K_{SS^*}^h\right)^T\left(D^h\right)^{-1}\right)\eta_{\cdot h} \\
&= \eta_{\cdot h}^T\left(D^h\right)^{-1}\eta_{\cdot h} - \eta_{\cdot h}^T\left(D^h\right)^{-1}K_{SS^*}^h\left(K_{S^*S^*}^h + \left(K_{SS^*}^h\right)^T\left(D^h\right)^{-1}K_{SS^*}^h\right)^{-1}\left(K_{SS^*}^h\right)^T\left(D^h\right)^{-1}\eta_{\cdot h} \\
&= \left\|\eta_{\cdot h}^T\left(D^h\right)^{-0.5}\right\|_2^2 + \left\|\eta_{\cdot h}^T\left(D^h\right)^{-1}K_{SS^*}^h L^{-T}\right\|_2^2, \qquad LL^T = \left(K_{S^*S^*}^h + \left(K_{SS^*}^h\right)^T\left(D^h\right)^{-1}K_{SS^*}^h\right)
\end{aligned}$$

This expression is computed at $O\left(n_y m^2 + m^3\right)$ complexity, hence scaling linearly with number of sites n_y. Similarly, the determinant

$$\left|\widehat{\mathrm{K}}^h\right| = \left|K_{SS^*}^h\left(K_{S^*S^*}^h\right)^{-1}\left(K_{SS^*}^h\right)^T + D^h\right| = \left|K_{S^*S^*}^h + \left(K_{SS^*}^h\right)^T\left(D^h\right)^{-1}K_{SS^*}^h\right|\left|K_{S^*S^*}^h\right|^{-1}\left|D^h\right|$$

is also calculated at the cost of $O\left(n_y m^2 + m^3\right)$, which brings the whole complexity of the full conditional sampler to $O\left(n_{\alpha_g} n_f\left(n_y m^2 + m^3\right)\right)$.

Nearest Neighbor Gaussian process

Full-conditional updater for H

If the latent factors were assigned NNGP prior, then the full-conditional distribution for $\boldsymbol{\eta}$ follows:

$$(\boldsymbol{\eta}|-)\sim N\left(\mu_\eta, \mathrm{U}_\eta\right), \qquad \mathrm{U}_\eta^{-1} = \widehat{\mathrm{K}}_{\mathrm{H}}^{-1} + \Lambda^T\Sigma^{-1}\Lambda \otimes I_{n_y}, \qquad \boldsymbol{\mu}_\eta = \mathrm{U}_\eta \mathrm{vec}(Z\Sigma^{-1}\Lambda)$$

Despite of the precision matrix U_η^{*-1} being sparse, neither its inverse, nor Cholesky decomposition are necessarily sparse. This effective negates all potential computational benefits due to sparsity in the $\widehat{\mathrm{K}}_{\mathrm{H}}^{-1}$. So, instead of sampling $\boldsymbol{\eta}$ directly, we obtain it as the permutation of $\boldsymbol{\eta}^*$, which could be obtained through following formulas

$$(\boldsymbol{\eta}^*|-)\sim N\left(\boldsymbol{\mu}_{\eta^*}, \mathrm{U}_{\eta^*}\right), \qquad \mathrm{U}_{\eta^*}^{-1} = P_{\eta^*\eta}\widehat{\mathrm{K}}_{\mathrm{H}}^{-1}P_{\eta^*\eta}^T + I_{n_y} \otimes \Lambda^T\Sigma^{-1}\Lambda, \qquad \boldsymbol{\mu}_{\eta^*} = \mathrm{U}_{\eta^*}\mathrm{vec}((Z\Sigma^{-1}\Lambda)^T)$$

Where the $P_{\eta^*\eta}$ is the transposition matrix that transforms $\boldsymbol{\eta}$ to $\boldsymbol{\eta}^*$: $\boldsymbol{\eta}^* = P_{\eta^*\eta}\boldsymbol{\eta}$. Now, the $\mathrm{U}_{\eta^*}^{-1}$ matrix has a special structure – if considering it as a block matrix with $n_y \times n_y$ blocks of size $n_f \times n_f$, all of its non-zero blocks are located at the same places as the non-zero elements of $\left(\widehat{\mathrm{K}}^h\right)$. So, the non-zero elements of $\mathrm{U}_{\eta^*}^{-1}$ are in the proximity of the diagonal, which allows for a sparse Cholesky factorization $\mathrm{U}_{\eta^*}^{-1} = L_{\eta^*}L_{\eta^*}^T$. Exact number of non-zero elements depends on configuration of sites and ordering, but it can be shown that for sites located at the vertices of a uniform square grid the number of non-zero elements would be of order $O\left(m n_f^2 n_y^{\frac{3}{2}}\right)$. Then, the random draw from the desired distribution $(\boldsymbol{\eta}^*|-)\sim N\left(\boldsymbol{\mu}_{\eta^*}, \mathrm{U}_{\eta^*}\right)$ can be obtained via following expressions:

$$\boldsymbol{\eta}^* = L_{\eta^*}^{-\mathrm{T}}\left(L_{\eta^*}^{-1}\mathrm{vec}((Z\Sigma^{-1}\Lambda)^T) + \boldsymbol{z}\right), \qquad \boldsymbol{z}\sim N\left(0_{n_y n_f \times 1}, I_{n_y n_f}\right)$$

The associated computational cost is mainly due to the sparse Cholesky decomposition and double left division of a $n_y n_f$-length vector to a $n_y n_f \times n_y n_f$ sparse triangular matrix.

In our Matlab implementation of this updater we use the Matlab's implementation of Cholesky factorization – function **chol**(). This function also provides an option to internally perform approximate minimum degree (AMD) permutation in order to get a sparser Cholesky factor. Unfortunately, we cannot report the exact algorithm that is used there, since is not publicly unclosed. We propose to keep this option on, since in our numerical experiments it allowed for an approximately 10-15% additional speed-up for the updater, although it is likely that for certain site configurations it would only generate unnecessary minor overheat (e.g. when all sites are on a single straight line). As a side remark, we would like to mention that applying this function directly to U_η^{-1} does not produce any reasonably sparse Cholesky factor due to heuristic nature of AMD algorithm, hence the block-ordered permutation to $\mathrm{U}_{\eta^*}^{-1}$ is indeed essential.

Full-conditional updater for α

Conditioning on H, spatial range parameters α_h are independent for $h = 1 \dots n_f$ and can be sampled one by one from the prior values α_g proportional to their conditional posterior probabilities. We follow the sampling scheme presented in Ovaskainen et al. (2016b), but exploit the special structure of NNGP-induced covariance matrices $\widehat{\mathrm{K}}^h$.

For each latent factor this scheme requires n_{α_g} calculations of the quadratic form $\eta_{\cdot h}^T \left(\widehat{\mathrm{K}}^h\right)^{-1} \eta_{\cdot h}$, and additionally n_{α_g} calculations of $\left|\widehat{\mathrm{K}}^h\right|$ that are shared among the latent factors.

$$\eta_{\cdot h}^T \left(\widehat{\mathrm{K}}^h\right)^{-1} \eta_{\cdot h} = \eta_{\cdot h}^T \left(\mathrm{I} - \widehat{\mathrm{A}}^h\right)^{-1} \widehat{\mathrm{D}}^h \left(\mathrm{I} - \widehat{\mathrm{A}}^h\right)^{-T} \eta_{\cdot h} = \left\| \eta_{\cdot h}^T \left(\mathrm{I} - \widehat{\mathrm{A}}^h\right)^{-1} \left(\widehat{D}^h\right)^{-0.5} \right\|_2^2, \qquad \left|\widehat{\mathrm{K}}^h\right| = \left|\widehat{\mathrm{D}}^h\right|$$

This first expression is computed at $O\left(n_y m\right)$ complexity, the second – just at $O\left(n_y\right)$ – hence the computation scales linearly with number of sites n_y. The construction process of matrices $\widehat{\mathrm{A}}^h$ and $\widehat{\mathrm{D}}^h$ takes $O\left(n_y m^3\right)$, but must be done only once for each α_g at the beginning of MCMC sampling. Hence, the resulted complexity of the full-conditional updater is $O\left(n_{\alpha_g} n_f n_y m\right)$.

Lemma 1: on full conditional updater of H for latent factors with GPP prior

Lemma: when each latent factor is assigned with Gaussian predictive process prior with marginal variance correction:

$$\eta_{\cdot h} \sim N\left(0_{n_y \times 1}, K^h\right)$$

$$K^h = K_{SS^*}^h \left(K_{S^*S^*}^h\right)^{-1} K_{S^*S}^h + D^h, \qquad D_{ij}^h = \delta_{ij}\left(1 - K_{s_i S^*}^h \left(K_{S^*S^*}^h\right)^{-1} K_{S^* s_i}^h\right), \qquad |S^*| = m$$

a random draw of the vector of all latent factors values $\eta_{ih}, \forall i = 1 \dots n_y, \forall h = 1 \dots n_f$ from its full conditional posterior distribution in HMSC can be obtained with computational cost of at most $\bar{O}\left(m n_f^2 n_y + m^3 n_f^3\right)$ flops.

Proof:

Then the joint prior for $\boldsymbol{\eta} = vec(\mathrm{H}) = \left[\eta_{\cdot 1}^T, \dots, \eta_{\cdot n_f}^T\right]^T$ is

$$\boldsymbol{\eta} \sim N\left(0_{n_y n_f \times 1}, W\right)$$

$$W = W_{12} W_{22} W_{21} + D$$

$$W_{12} = diag\left(K_{SS^*}^1, \dots, K_{SS^*}^{n_f}\right), \qquad W_{21} = W_{12}^T, \qquad W_{22} = diag\left(K_{S^*S^*}^1, \dots, K_{S^*S^*}^{n_f}\right)$$

$$D = diag(D^1, \dots, D^{n_f})$$

Then the conditional distribution of $\boldsymbol{\eta}$ would be

$$\boldsymbol{\eta} \sim N(\boldsymbol{\mu}_\eta, \mathrm{U}_\eta)$$

$$\mathrm{U}_\eta^{-1} = W^{-1} + \Lambda^T \Sigma^{-1} \Lambda \otimes I_{n_y}$$

$$\boldsymbol{\mu}_\eta = \mathrm{U}_\eta \mathrm{vec}(\mathrm{S}^r \Sigma^{-1} \Lambda)$$

However, in case of large n_y direct sampling from this distribution is problematic due to the need to invert and decompose the large dense matrix U_η^{-1}. Instead the special form of W can be exploited for enhanced performance.

First using the Woodbury identity

$$W^{-1} = (W_{12} W_{22} W_{21} + D)^{-1} = D^{-1} - D^{-1} W_{12} (W_{22} + W_{21} D^{-1} W_{12})^{-1} W_{21} D^{-1}$$

Then denoting by $M = W_{22} + W_{21} D^{-1} W_{12}$, and by $A = \Lambda^T \Sigma^{-1} \Lambda \otimes I_{n_y} + D^{-1}$,

$$\left(W^{-1} + \Lambda^T \Sigma^{-1} \Lambda \otimes I_{n_y}\right)^{-1} = (A - D^{-1} W_{12} M^{-1} W_{21} D^{-1})^{-1}$$

Which can be further expanded with Woodbury identity

$$(A - W_{12} M^{-1} W_{21} D^{-1})^{-1} = A^{-1} + A^{-1} D^{-1} W_{12} (M - W_{21} D^{-1} A^{-1} D^{-1} W_{12})^{-1} W_{21} D^{-1} A^{-1}$$

Matrix $F = M - W_{21} D^{-1} A^{-1} D^{-1} W_{12}$ has the size $n_k n_f \times n_k n_f$, and its inverse F^{-1} and its Cholesky decomposition $L_{F^{-1}}$ could be easily computed once the number of Gaussian predictive process knots is relatively small.

The inverse matrix A^{-1} can be also effectively calculated due to its special structure. Let's denote by P the commutation matrix between $\Lambda^T \Sigma^{-1} \Lambda \otimes I_{n_y}$ and $I_{n_y} \otimes \Lambda^T \Sigma^{-1} \Lambda$, so that $\Lambda^T \Sigma^{-1} \Lambda \otimes I_{n_y} = P\left(I_{n_y} \otimes \Lambda^T \Sigma^{-1} \Lambda\right) P^T$. This matrix always exists and $P^T P = P P^T = I_{n_y n_f}$. Further, $P^T R P$ and $P^T R P$ are diagonal matrices if R is diagonal. Then

$$A = \Lambda^T \Sigma^{-1} \Lambda \otimes I_{n_y} + D^{-1} = P\left(I_{n_y} \otimes \Lambda^T \Sigma^{-1} \Lambda\right) P^T + P P^T D^{-1} P P^T = P\left(I_{n_y} \otimes \Lambda^T \Sigma^{-1} \Lambda + P^T D^{-1} P\right) P^T =$$

$$= P\left(diag\left(\Lambda^T \Sigma^{-1} \Lambda + \widetilde{D}^1, \ldots, \Lambda^T \Sigma^{-1} \Lambda + \widetilde{D}^{n_y}\right)\right) P^T$$

Where $\widetilde{D}^i$ are $n_f \times n_f$ diagonal matrices, such that $P^T D^{-1} P = diag\left(\widetilde{D}^1, \ldots, \widetilde{D}^{n_y}\right)$. Then

$$A^{-1} = \left(P\left(diag\left(\Lambda^T \Sigma^{-1} \Lambda + \widetilde{D}^1, \ldots, \Lambda^T \Sigma^{-1} \Lambda + \widetilde{D}^{n_y}\right)\right) P^T\right)^{-1} = P^T\left(diag\left(\left(\Lambda^T \Sigma^{-1} \Lambda + \widetilde{D}^1\right)^{-1}, \ldots, \left(\Lambda^T \Sigma^{-1} \Lambda + \widetilde{D}^{n_y}\right)^{-1}\right)\right) P$$

which requires only n_y inversions of $n_f \times n_f$ matrices that are typically small. Furthermore matrix A^{-1} is sparse with at most $n_y n_f^2$ non-zeros elements. Finally

$$L_{A^{-1}} = chol(A^{-1}) = P^T\left(diag\left(chol\left(\left(\Lambda^T \Sigma^{-1} \Lambda + \widetilde{D}^1\right)^{-1}\right), \ldots, chol\left(\left(\Lambda^T \Sigma^{-1} \Lambda + \widetilde{D}^{n_y}\right)^{-1}\right)\right)\right) P$$

also requires only computing the Cholesky decomposition of n_y small $n_f \times n_f$ matrices and $L_{A^{-1}}$ is sparse with at most $n_y \frac{n_f(n_f - 1)}{2}$ non-zeros elements.

Finally, we get that

$$\boldsymbol{\eta} \sim N(\boldsymbol{\mu}_\eta^*, \mathrm{U}_\eta^*)$$

Could be sampled as

$$\boldsymbol{\eta} = A^{-1} \mathrm{vec}(\mathrm{S}\Sigma^{-1}\Lambda) + (A^{-1} D^{-1} W_{12} L_{F^{-1}})\left((A^{-1} D^{-1} W_{12} L_{F^{-1}})^T \mathrm{vec}(\mathrm{S}\Sigma^{-1}\Lambda)\right) + L_{A^{-1}} \boldsymbol{z}_1 + (A^{-1} D^{-1} W_{12} L_{F^{-1}}) \boldsymbol{z}_2$$

$$\boldsymbol{z}_1 \sim N\left(0_{n_y n_f \times 1}, I_{n_y n_f}\right), \qquad \boldsymbol{z}_2 \sim N\left(0_{m n_f \times 1}, I_{m n_f}\right)$$

This has the numerical complexity of $\bar{O}\left(\max\left(m n_f^2 n_y, m^3 n_f^3\right)\right)$ flops, so scaling linearly as the number of sites.

Posterior predictive distribution

Once the draws from the posterior of HMSC model parameters have been acquired, they can be used for making predictions at any location, where the values of covariates are known. We denote the desired prediction location by $\boldsymbol{s}_*$, the set of covariates included to the fixed effects component of the HMSC model at that location by $\boldsymbol{x}_*$, and the vector of predicted outcomes by $\boldsymbol{y}_*$. We denote the set of all model parameters, specified in the corresponding section above by θ. Then

$$p(\boldsymbol{y}_*|\boldsymbol{x}_*, Y) = \int_{\theta} p(\boldsymbol{y}_*|\boldsymbol{x}_*, \theta) p(\theta|Y) d\theta \approx \frac{1}{Q}\sum_{q=1}^{Q} p\left(\boldsymbol{y}_*|\boldsymbol{x}_*, \theta^{(q)}\right)$$

Where $\left\{\theta^{(1)}, \dots, \theta^{(Q)}\right\}$ is the set of posterior samples.

$$p\left(\boldsymbol{y}_*|\boldsymbol{x}_*, \theta^{(q)}\right) = \int_{\boldsymbol{l}_*} p\left(\boldsymbol{y}_*|\boldsymbol{l}_*, \boldsymbol{\sigma}^{(q)}\right) p\left(\boldsymbol{l}_*|\boldsymbol{x}_*, \theta^{(q)}\right) d\boldsymbol{l}_* = \int_{\boldsymbol{\eta}_*} p\left(\boldsymbol{y}_*|\mathrm{B}_{(q)}^T \boldsymbol{x}_* + \Lambda_{(q)}^T \boldsymbol{\eta}_*, \boldsymbol{\sigma}^{(q)}\right) p\left(\boldsymbol{\eta}_*|\mathrm{H}_{(q)}, \boldsymbol{\alpha}_{(q)}\right) d\boldsymbol{\eta}_*$$
$$\approx \frac{1}{R}\sum_{r=1}^{R} p\left(\boldsymbol{y}_*|\mathrm{B}_{(q)}^T \boldsymbol{x}_* + \Lambda_{(q)}^T \boldsymbol{\eta}_*^{(r)}, \boldsymbol{\sigma}^{(q)}\right),$$

where $\left\{\boldsymbol{\eta}_*^{(1)}, \dots, \boldsymbol{\eta}_*^{(R)}\right\}$ are samples from the conditional distribution $p\left(\boldsymbol{\eta}_*|\mathrm{H}_{(q)}, \boldsymbol{\alpha}_{(q)}\right)$, which governs the latent factors values in $\boldsymbol{s}_*$ given the realization H_q in training locations S. From now on we would drop the index of posterior sample q for the clarity of notation. This conditional distribution factorizes across the latent factors

$$p(\boldsymbol{\eta}_*|\mathrm{H}, \boldsymbol{\alpha}) = \prod_{h=1}^{n_f} p(\eta_{h*}|\eta_{\cdot h}, \alpha_h),$$

which enables to obtain conditional samples of the joint $\boldsymbol{\eta}_*$ via sampling from univariate conditional distributions for different latent factors h. This allows to retain linear asymptotic complexity in predictive distribution with respect to the number of latent factors n_f in the HMSC. Formulas for efficient sampling from univariate conditional variance-corrected GPP and NNGP distributions follow the original strategies (Finley et al. 2009, Datta et al. 2016a).

Details on Australian plants case study

The data originate from the Victorian Biodiversity Atlas (VBA) (https://www.environment.vic.gov.au/biodiversity/victorian-biodiversity-atlas), which is a state database that collaborates with the Atlas of Living Australia (http://www.ala.org.au). The subset of the VBA used in this study involves the occurrences of 1237 herbaceous species, at 30,955 sampling locations within the State of Victoria, Australia (Fig. 2A), for which presence-absence were recorded. The data were collected in years 1984-2014 on sampling plots of 3900 m^2, The number of unique survey teams involved in the collection of these data is not known accurately, but is in the order of 200-300. The dataset combines survey data undertaken for a range of purposes the predominant being:

1. Ecosystem inventory, circumscription and mapping
2. Characterizing the habitats of species of management interest
3. Documenting and describing land subject to development or land-use change

Consequently, the data is biased towards sampling public lands, typically less suitable for agriculture and peri-urban areas.

We selected four environmental covariates that were considered potentially important to vegetation and plant distribution and were not strongly correlated. These measure:

1. Climatic conditions – Mean maximum temperature in January (the hottest and driest month in south eastern Australia), developed using ANUCLIM (Houlder et al 2000). See Appendix S1: Fig. S1A.
2. Hydrology and landscape position – This a summed and normalized set of 'vertical distance above stream' calculations (Conrad et. al. 2015) for seven different channel networks, each of which satisfy seven separate, monotonically increasing, flow accumulation thresholds (based on catchment size weighted by catchment rainfall). See Appendix S1: Fig. S1B.
3. Soil properties - Here we used the radioelement count of thorium as a general proxy for soil type. Radiometric data is related to soil depth, soil texture and nutrition particularly in surficial landscapes. See Read et al. (2018) and Appendix S1: Fig. S1C.
4. Solar radiation and anisotrophic heating. These data have been derived from the transformation of a digital elevation model to indicate the relative level of terrain illumination when the sun is at 270 degrees (North-West) and 40 degrees above the horizon.

Pearson correlation between selected environmental covariates at observed sites				
	climatic conditions	water proximity	soil properties	local slope
climatic conditions	1	-0.47	-0.09	-0.19
water proximity	-0.47	1	0.38	0.15
soil properties	-0.09	0.38	1	0.08
local slope	-0.19	0.15	0.08	1

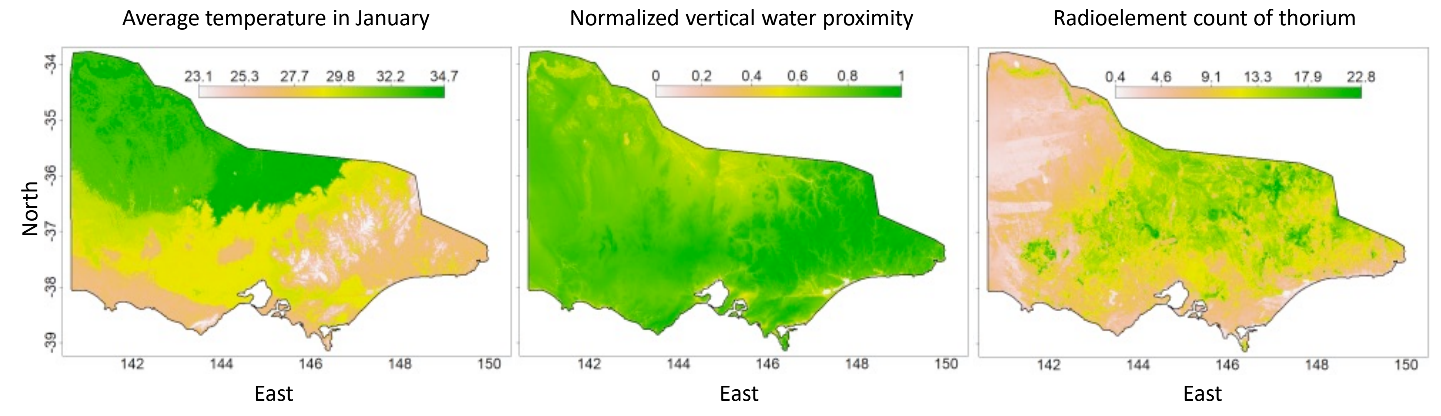

Figure S1. Spatial distribution of environmental covariates included to the model.

We also included available information on 9 species traits as binary indicator variables, describing whether the species (1) is annual or perennial, (2) is pollinated by abiotic or biotic means, (3-4) has propagules that are dispersed by wind, invertebrates, or another agent, (5) forms a seed bank that typically persists for two or more years, and is considered vulnerable to or tolerant of (6) fire, (7) prolonged snow cover, (8) protracted waterlogging, or (9) salinity. These traits were selected from a much larger list of expert-provided traits that potentially govern the species distribution in the studied community. The particular choice of those included to the model was governed by a) amount of collinearity between different available traits and b) availability of trait values for all the studied species.

Details on model convergence

As described in the main text, we fitted all models with 10,000 MCMC steps, out of which we discarded the first 2,000 steps as burn in. We thinned the remaining samples by 10, resulting in 800 posterior draws. To examine the convergence of the MCMC chains, we repeated model fitting 40 times, randomly selecting initial parameter values from the prior distribution. We assessed the quality of mixing by calculating the effective sample sizes (ESS) and potential scale reduction factors

(PSRF) for the model parameters (Gelman and Rubin 1992). However, as the prior of Bhattacharya and Dunson (2011) leads to non-identifiable parameters H and Λ, and the number of unique entries in $\Omega = \Lambda^{\mathrm{T}}\Lambda$ is extremely high for model fitted to all data, we restricted our calculations to the B parameters and a randomly chosen 40×40 symmetric submatrix of association matrix $R = \mathrm{cov2cor}(\Omega + I_{n_s})$, as those are fundamental in the ecological applications and generally representative for overall model mixing. Hence, for each variant of fitted model we stacked the 40 chains, each containing 800 draws of B and R parameters, and calculated the ESS and PSRF with `effectiveSize()` and `gelman.diag()` functions implemented in `coda` R package. To reduce still enormously high number of quantities, we calculated a single quantity for each model variant – the 5% quantile of the ESS and the 95% quantile of the PSRF point estimates among the β_{kj} or $R_{jj'}$ parameters in this model variant. The resulted values are summarized in the Appendix S1: Fig. S2, Fig. S3, Fig. S4, Fig. S5.

As is clearly seen from the visualization of the effective sample sizes and potential scale reduction factors, the algorithm with selected number of steps and thinning demonstrated generally adequate mixing for numbers of training sites up to 1600 and got significantly worse when the number of sites further increased. The drop is especially pronounced for the elements of association matrix $R_{jj'}$. On the other hand, the number of species exhibited opposite impact – with higher number of species the effective sample sizes were generally higher than with low number of species. Perhaps somewhat unexpectedly, the mixing in model fits with high number of training sites got insufficient also for non-spatial model. These results suggest that to keep the results of Bayesian analysis properly valid (especially concerning uncertainty quantifications), the number of samples or thinning for models fitted to large data must be increased, which raise the right parts of the expected computation times that are shown in the Fig. 1A-F.

Despite of the fact that the bigger model indicated insufficient mixing, in terms of predictive performance our results were quite stable between different chains and qualitatively repeated the results averaged over the chains. This provides an empirical confirmation that the predictive performance is expected to behave as reported in Fig. 1 and would not considerably depend on initial starting position of MCMC chains. Appendix S1: Fig. S6 and Fig. S7 extend the Fig. 1 GHI and Fig. 1 JKL correspondingly by presenting the predictive measures calculated for each of the independent chains. The visualization of performance distribution for each model variant is constructed using `beanplot` function with standard smoothing parameters from `beanplot` R package.

Based on the complications with mixing that we have encountered when fitting models to the larger datasets, we would like to summarize that the block Gibbs sampling algorithm, presented by Ovaskainen et al. (2017) is insufficiently efficient for modelling big datasets, at least when the outcomes are binary. One potential bottleneck is due to known inefficiencies of the data augmentation of Albert and Chib (1993) that is used for dealing with binary data, which leads to slow mixing for unbalanced outcomes, which probability is close to zero or one. The fundamental problem comes from the great mismatch of marginal posterior and the conditional distribution given the augmented data. Some recent work has been conducted aiming to efficiently deal with this issue (Duan et al. 2017), specifically to "widen" the conditional distribution at the cost of introducing a rejection probability. However, its utility has been demonstrated on a significantly simpler models and transition of those results to HMSC is not devised yet. Another opportunity, which currently seems more promising in our opinion, is to investigate how combination of marginal representation of HSMC's latent liabilities as a Gaussian process could be coupled with approximate methods for dealing with non-Gaussian observations (e.g. Laplace approximation, expectation propagation, variational inference). While the resulted GP would be of dimension $n_y n_s$, which in case of our biggest training dataset is over $1.6 \cdot 10^6$ and prohibits full GP fitting approach, the special structure of GP's covariance matrix induced by HMSC structure provides opportunities for much more efficient solutions. For the GPP model, such method would have the same flavor as the approximate inference methods for Gaussian process regression/classification (Hensman et al. 2015). For the NNGP model, such approaches seem to be conceptually more challenging as the matrix sparsity there is tricky to utilize in combination with other marginalized model components. Thus, we believe that the first steps in that direction should be in developing an extension of the collapsed NNGP method, proposed by Finley et al. (2019), which extension would additionally marginalizing out the fixed effects and be applicable to non-Gaussian residuals. To sum up, we would like to mark this research question as a potential area of interest for statisticians and machine learner researchers, specializing in developing methods for multivariate Bayesian data analysis.

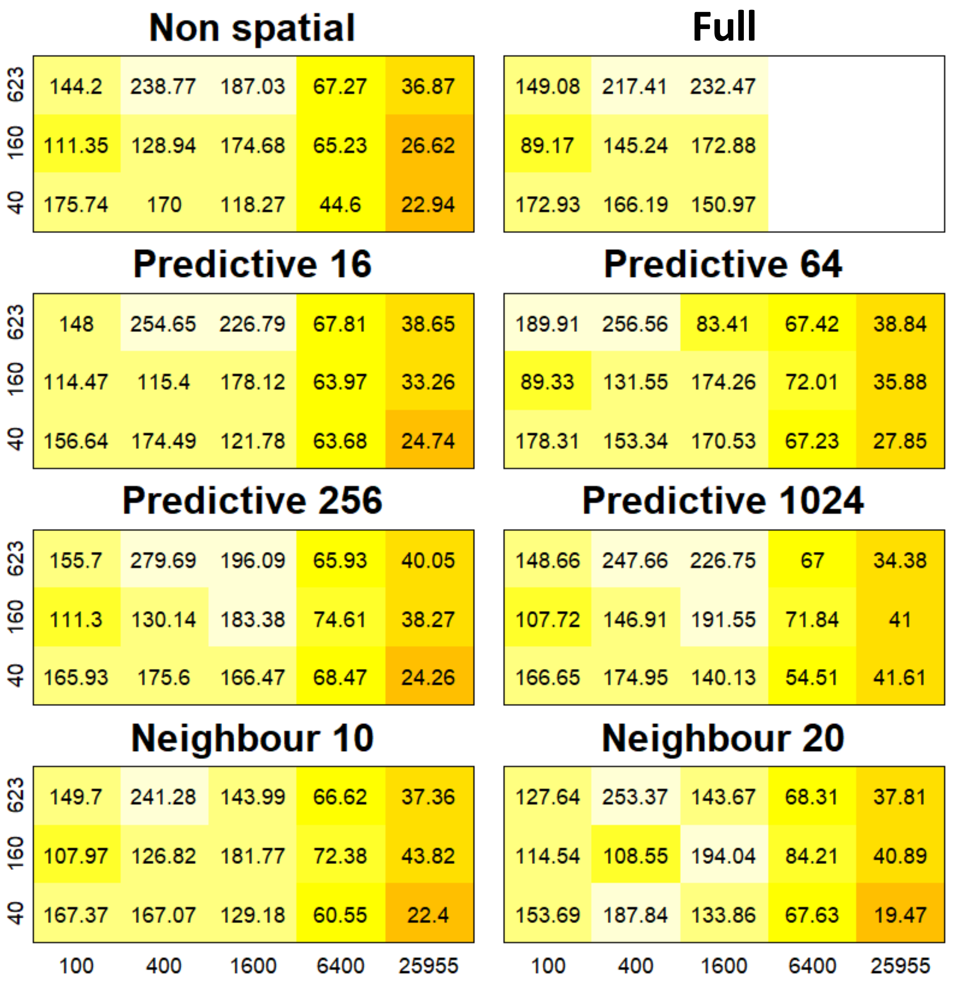

Figure S2. Visualization of effective sample sizes for covariate responses based on multiple fitted independent chains for each of model variants. The numbers depict the 5% quantiles among the β_{kj} parameters.

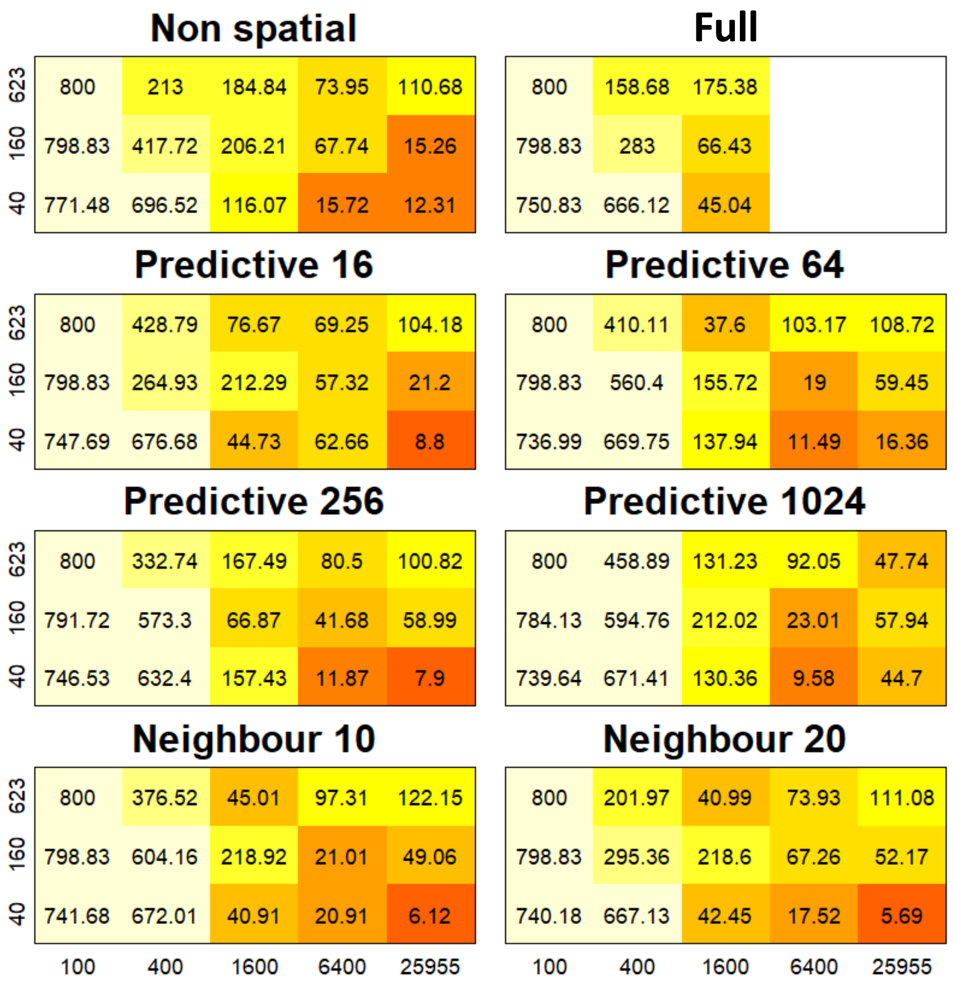

Figure S3. Visualization of effective sample sizes for association matrices based on multiple fitted independent chains for each of model variants. The numbers depict the 5% quantiles among the off-diagonal $R_{jj'}$ parameters.

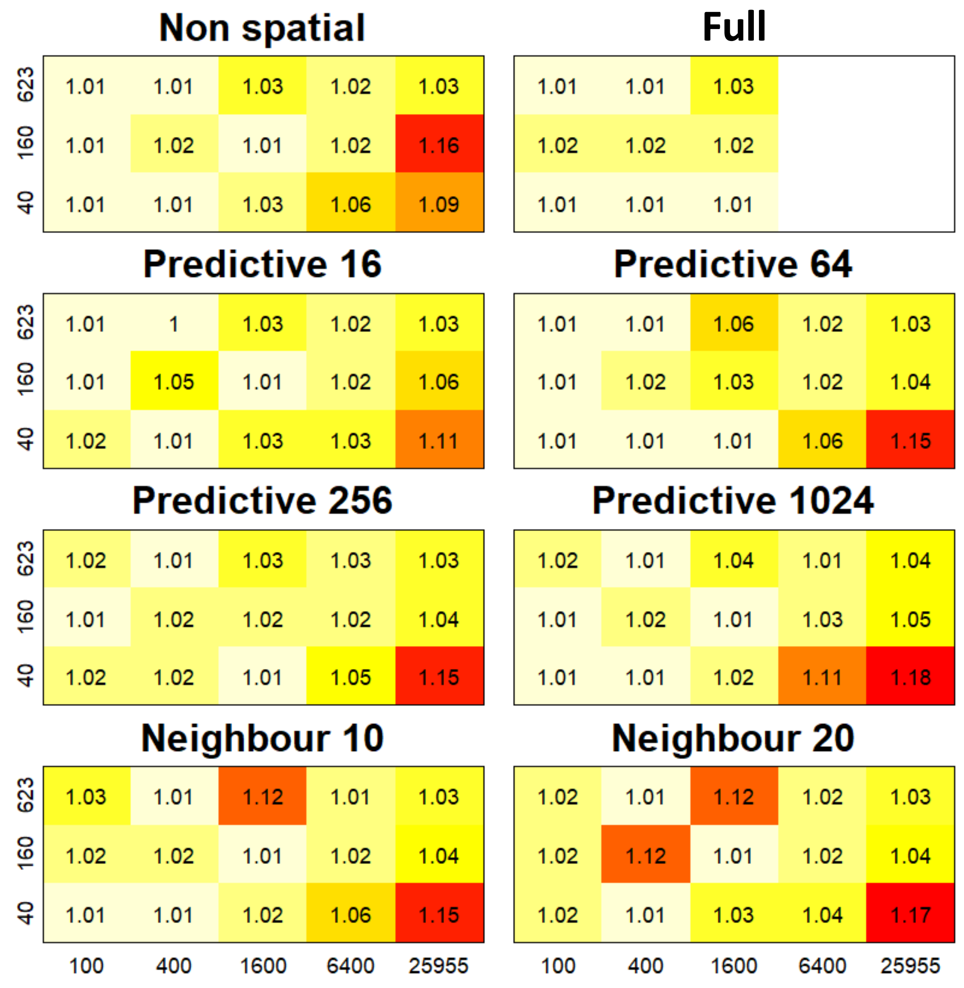

Figure S4. Visualization of PSRF for covariate responses based on multiple fitted independent chains for each of model variants. The numbers depict the 95% quantiles among the β_{kj} parameters.

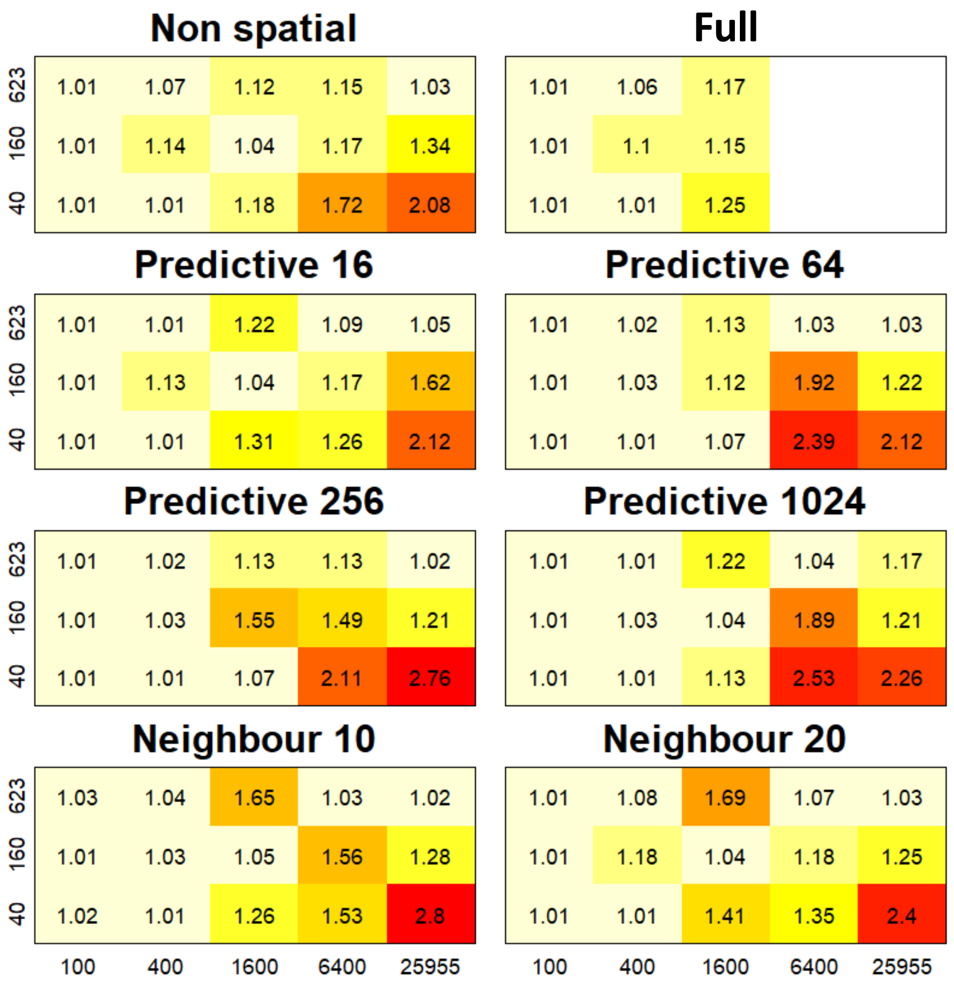

Figure S5. Visualization of PSRF for association matrices based on multiple fitted independent chains for each of model variants. The numbers depict the 95% quantiles among the off-diagonal $R_{jj'}$ parameters.

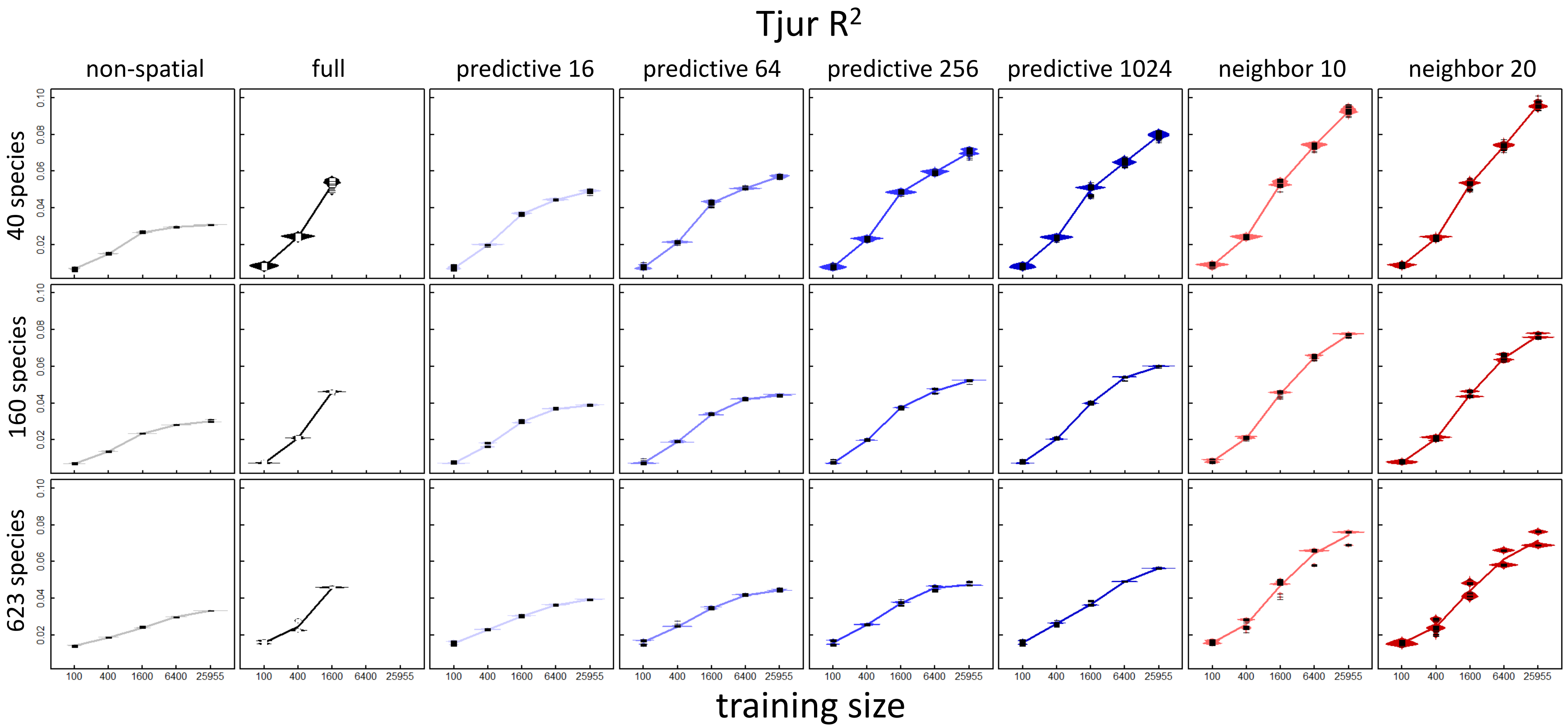

Figure S6. Extension of Fig. 1EFG visualizing the predictive Tjur R^2 for each independent chain (black horizontal stripes). The selected coloring follow Fig 1.

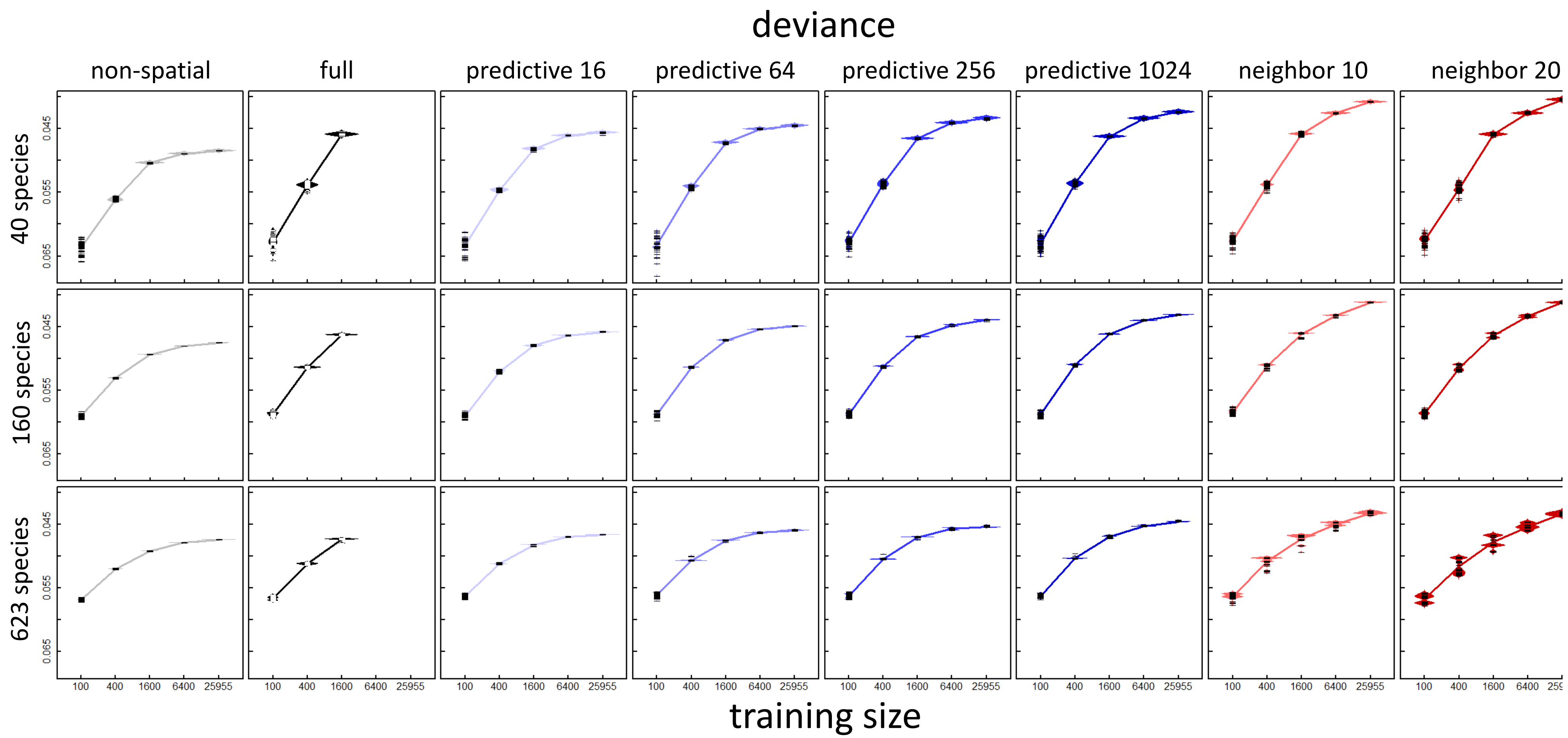

Figure S7. Extension of Fig. 1HIJ visualizing the predictive deviance for each independent chain (black horizontal stripes). The selected coloring follow Fig 1.

References

Albert, J. H., and S. Chib. 1993. Bayesian analysis of binary and polychotomous response data. Journal of the American Statistical Association **88**:669-679.

Banerjee, S., A. E. Gelfand, A. O. Finley, and H. Sang. 2008. Gaussian predictive process models for large spatial data sets. Journal of the Royal Statistical Society. Series B: Statistical Methodology **70**:825-848.

Bhattacharya, A., and D. B. Dunson. 2011. Sparse Bayesian infinite factor models. Biometrika **98**:291-306.

Datta, A., S. Banerjee, A. O. Finley, and A. E. Gelfand. 2016a. Hierarchical Nearest-Neighbor Gaussian Process Models for Large Geostatistical Datasets. Journal of the American Statistical Association **111**:800-812.

Datta, A., S. Banerjee, A. O. Finley, and A. E. Gelfand. 2016b. On nearest-neighbor Gaussian process models for massive spatial data. Wiley Interdisciplinary Reviews: Computational Statistics **8**:162-171.

Duan, L. L., J. E. Johndrow, and D. B. Dunson. 2017. Calibrated data augmentation for scalable Markov Chain Monte Carlo. arxive.

Finley, A. O., A. Datta, B. D. Cook, D. C. Morton, H. E. Andersen, and S. Banerjee. 2019. Efficient algorithms for Bayesian nearest neighbor Gaussian processes. Journal of Computational and Graphical Statistics:1-14.

Finley, A. O., H. Sang, S. Banerjee, and A. E. Gelfand. 2009. Improving the performance of predictive process modeling for large datasets. Comput Stat Data Anal **53**:2873-2884.

Gelman, A., and D. B. Rubin. 1992. Inference from iterative simulation using multiple sequences. Statistical Science **7**:457-472.

Guinness, J. 2018. Permutation and Grouping Methods for Sharpening Gaussian Process Approximations. Technometrics **60**:415-429.

Hensman, J., A. Matthews, and Z. Ghahramani. 2015. Scalable variational Gaussian process classification. Journal of Machine Learning Research **38**:351-360.

Lopes, H. F., and M. West. 2004. Bayesian model assessment in factor analysis. Statistica Sinica **14**:41-68.

Ovaskainen, O., N. Abrego, P. Halme, and D. Dunson. 2016a. Using latent variable models to identify large networks of species-to-species associations at different spatial scales. Methods in Ecology and Evolution **7**:549-555.

Ovaskainen, O., D. B. Roy, R. Fox, and B. J. Anderson. 2016b. Uncovering hidden spatial structure in species communities with spatially explicit joint species distribution models. Methods in Ecology and Evolution **7**:428-436.

Ovaskainen, O., G. Tikhonov, A. Norberg, F. G. Blanchet, L. Duan, D. Dunson, T. Roslin, and N. Abrego. 2017. How to make more out of community data? A conceptual framework and its implementation as models and software. Ecology Letters **20**:561-576.

Read, C. F., D. H. Duncan, C. Y. C. Ho, M. White, and P. A. Vesk. 2018. Useful surrogates of soil texture for plant ecologists from airborne gamma-ray detection. Ecol Evol **8**:1974-1983.

Ren, Q., and S. Banerjee. 2013. Hierarchical factor models for large spatially misaligned data: a low-rank predictive process approach. Biometrics **69**:19-30.

Stein, M. L., Z. Chi, and L. J. Welty. 2004. Approximating likelihoods for large spatial data sets. Journal of the Royal Statistical Society: Series B (Statistical Methodology) **66**:275-296.

Taylor-Rodriguez, D., A. O. Finley, A. Datta, C. Babcock, H.-E. Andersen, B. D. Cook, D. C. Morton, and S. Banerjee. 2018. Spatial Factor Models for High-Dimensional and Large Spatial Data: An Application in Forest Variable Mapping. ArXiv e-prints.

Tikhonov, G., N. Abrego, D. Dunson, and O. Ovaskainen. 2017. Using joint species distribution models for evaluating how species-to-species associations depend on the environmental context. Methods in Ecology and Evolution:443-452.

Vecchia, A. V. 1992. A New Method of Prediction for Spatial Regression Models with Correlated Errors. Journal of the Royal Statistical Society. Series B (Methodological) **54**:813-830.

Zhou, M., L. Li, D. Dunson, and L. Carin. 2012. Lognormal and gamma mixed negative binomial regression. Proceedings of the International Conference on Machine Learning **2012**:1343-1350.
